# A new method for assessing plant lodging and the impact of management options on lodging in canola crop production

**DOI:** 10.1038/srep31890

**Published:** 2016-08-24

**Authors:** Wei Wu, Bao–Luo Ma

**Affiliations:** 1 College of Agronomy, Northwest A&F University, Yangling 712100, Shaanxi, China; 2 Ottawa Research and Development Centre, Agriculture and Agri–Food Canada, 960 Carling Ave, Ottawa, ON, K1A 0C6, Canada

## Abstract

Lodging, defined as the permanent displacement of aboveground parts, is a common problem to cause yield loss, deterioration in seed quality and difficult to harvest in canola (*Brassica napus L.*) crop production. This study aimed to develop a method for assessing crop lodging, to examine how agronomic practices affected the relationships between root lodging and electrical capacitance traits. Canola plants were more susceptible to root lodging than stem lodging. The electrical measurements were more closely related with anchorage strength (S_p_) than stem bending strength (S_s_). Among the three electrical measurements, the root capacitance (C) displayed the most consistent and significant relationships with S_p_ in all three field experiments (R^2^ = 0.88–0.56; P ≤ 0.01). This study indicates that the risk of lodging can be reduced by using appropriate management practices and variety selection. Enhancing root S_p_ was advocated as a priority over enhancing stem S_s_ in cultivar improvement. Electrical measurements, especially of root C, can be considered as a non–invasive technique that could partially replace the intrusive methods used for the *in situ* assessment of lodging resistance among various agronomic practices or can be applied in breeding programs for selecting genotypes with high yield potentials and strong S_p_ values.

Canola (*Brassica napus L.*) is a high value crop that is used as vegetable oil for human consumption as well as renewable biodiesel energy, and its meal as animal protein. Under favorable environmental conditions, canola has a high yielding potential. Thus, canola has become a major cash crop worldwide, and is widely grown in Canada, China, India, part of USA, and the European Union countries. Canola production is still less common in some regions of the world, such as in eastern Canada, mainly due to lacking crushing facilities and technology support, which make canola production economically unattractive to farmers. Therefore, with increasing interest in growing canola currently in the world^1^,^2^,^3^, development of appropriate agronomic practices that can be adopted by farmers is urgently needed^4^.

Lodging refers to the permanent displacement of aboveground portions of crops from their vertical stance due to stem buckling (stem lodging) or failure of the root–soil anchorage system (root lodging). Lodging is a common phenomenon in canola production and is the main constraint for increasing canola yields under favorable weather condition^5^,^6^,^7^,^8^.

Plant lodging can be classified as root lodging and stem lodging^9^. Modern breeding programs have selected for varieties with more rigid stems to resistant the crop lodging and high seed yields due to the improvement in harvest index and biomass. This breeding strategy would make stem buckling at a basal internode relatively uncommon in modern genotypes. Instead, this strategy leads to canola plants with greater self–weight that is more prone to root lodging^9^,^10^,^11^. Thus, root anchorage strength (S_p_) should be targeted and studied as an essential parameter. In fact, an individual plant’s resistance to lodging not only depends on its genetic characteristics^12^,^13^, but is also related to agronomic management practices the plants encountered in their development for the production of thicker stems and stronger root systems^14^,^15^,^16^.

It is well-known that luxuriant growth under high plant densities and/or excessive N application in cereal crops can substantially increase the risk to crop lodging^8^,^14^,^15^,^16^. This is because the dramatic increase in stem length, which represents the level of the lodging-inducing torque, under those circumstances. Some morphological parameters of basal internodes, such as stem diameter and wall thickness in barley, oat and rice significantly influence flexural rigidity, and thereby alter the stem lodging^5^,^12^. Chemical composition, such as cellulose, lignin, silica and structural carbohydrate contents in the basal internodes is responsible for culm rigidity and significantly influences the breaking resistance of cereal stems^5^,^13^,^16^. Recently, great attention has been focused on root system and its related mechanics of root anchorage, as most cereal crops are more likely to undergo root lodging than stem lodging^9^,^10^,^11^. Root anchorage in the soil medium is determined by the characteristics of the root system, and therefore root characteristics are important indicators for root lodging resistance^10^.

In a lodged cereal crop, a decrease in photosynthetic ability and biomass production has been reported in several studies because of its suppression in transporting of water and nutrients through the xylem and photosynthetic assimilates via phloem^5^,^8^. Multiple studies on rice, wheat and oats have shown that lodging can decrease grain yield and quality^4^,^5^. In addition, lodging can cause problems for harvest operations, increasing the demand for grain drying and, consequently, production costs^8^.

Integrated agronomic practices, including improved irrigation regime (such as subsurface drip irrigation, SDI), optimum planting date, balanced fertilizer management and variety selection, are important strategies for improving crop productivity and resource use efficiency^2^,^3^,^17^,^18^,^19^,^20^. All of these management options can alter soil water and nutrient uptake or even the agro–ecosystem conditions and agronomic performance, and then influence the phenology and the critical stage at which the sensitivity to crop lodging occurs^2^,^17^,^19^. Although it is clear that these agronomic practices can significantly affect the morphological characteristics of shoot and root distributions^5^, few studies have been published regarding the direct influences of agronomic practices on the lodging resistance of canola plants. Simulation models relating crop plant lodging and other biomechanical properties have been developed. Crook and Ennos^11^ proposed “safety factors” for relating shoot and anchorage failure, respectively by comparing the strengths of the stem and root structures with the loads they must bear. This method predicts the relative degrees of susceptibility of plants to anchorage failure and stem buckling, which are useful for assessing plant stability but do not consider the effects of wind. Baker *et al*.^21^ developed a more complex model that further considered aerodynamics, and regarded the wind–induced bending moment as the dominant factor controlling lodging. The model has been further revised according to the spatial non–uniformities between plants and their temporal changes^22^.

Those sophisticated models are promising. However, the time–consuming measurements and the requirement for biomechanical knowledge make the application of these models difficult by smallholders, agronomists and plant breeders, although agricultural engineers with biomechanical knowledge can easily use these models^23^. This is why lodging susceptibility ratings for genotypic differences, management options and environmental factors are frequently based upon observations of the actual lodging in the field. It only provides a rough estimation for lodging resistance evaluation if crop lodging occurs in a real situation and otherwise useless if crop lodging does not happen. New instrumentations and simple methods are needed to rapidly assess these biomechanical traits *in situ*. The primary objective of this study was therefore to develop a non–invasive method for assessing root S_p_, which is the most important parameter for improving lodging resistance.

Some controlled studies indicate the feasibility of using electrical measurements, including the root capacitance (C) and root impedance (Z), to estimate the fine root morphological traits in terms of surface area (A), root length (L) and volume (V), based on their linear relationships^24^,^25^,^26^,^27^. This particular method only requires an LCR bridge meter containing two electrodes, one placed at the plant base and the other placed in the surrounding plant growth medium. Each root section can be considered as an axially symmetric cylindrical condenser^28^. The epidermal membranes of the roots are believed to act as electrical insulators separating two conductive elements, the soil solution on the outer part of the membrane and the internal root medium^28^. The conceptual and theoretical models of root C and root Z that originated from Dalton, Aubrecht *et al*.^28^,^29^ are illustrated in more detail in Appendix I.

We hypothesize that canola plants with higher root C readings generally have superior root characteristics and subsequently construct a stronger S_p_ (please see Appendix I for the hypothesis and its theoretical explanation). Thus, the main objectives of this study are to (i) establish the relationships between electrical measurements and S_p_, and (ii) develop a new method for assessing lodging resistance. The results from this study will help us develop a rapid indicator for assessing lodging resistance among various agronomic practices. This technique could be applied in breeding programs for improving genotypes with strong S_p_ while sustaining a high–yield potential. The biomechanical properties of three canola varieties subjected to various agronomic practices are characterized under field experimental settings, which have provided a perfect platform for assessing the feasibility of using root electrical measurements to evaluate crop lodging parameters. This knowledge serves as the criteria for determining whether a plant is more susceptible to shoot or root lodging, and it could be used for implementing the best management options for mitigating lodging risks under high yielding conditions.

## Results

### Experiment I

#### Mechanical measurements and seed yield

**Figure 1:**
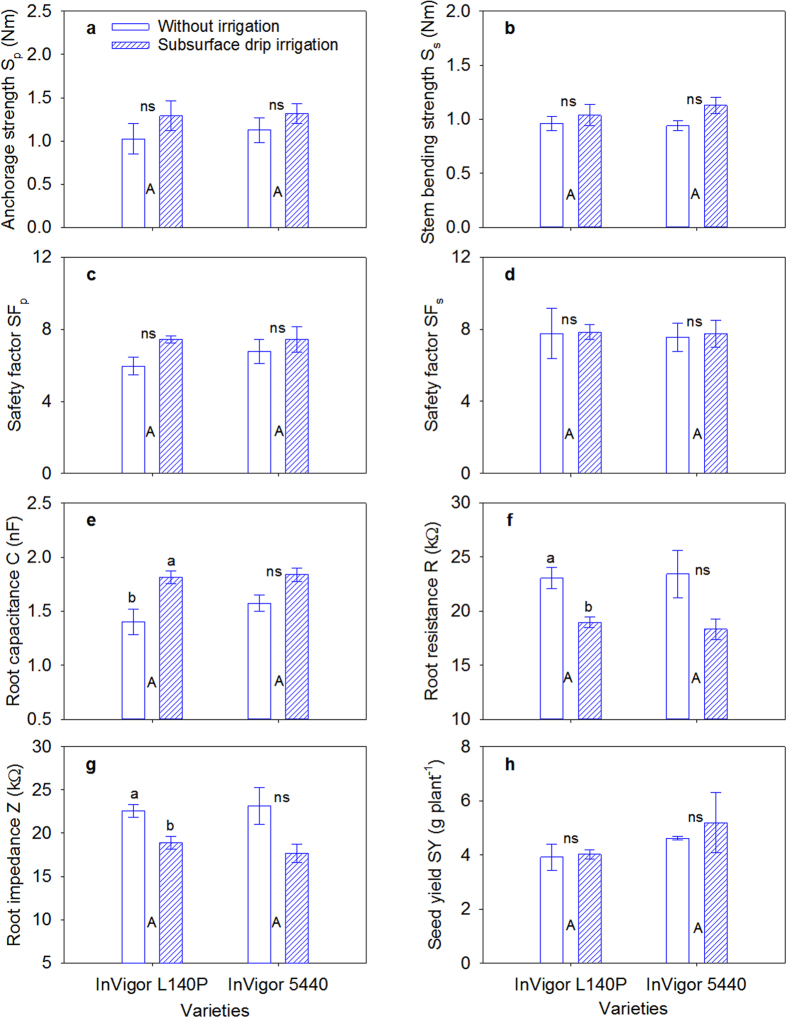
Effect of irrigation regime on (**a**) anchorage strength, (**b**) stem bending strength, (**c**) root safety factor SF_p_, (**d**) stem safety factor SF_s_, (**e**) root capacitance, (**f**) root resistance, (**g**) root impedance and (**h**) seed yield between the two varieties of canola. Vertical bars above mean values indicate standard error of three replications. Means with different small alphabetical letters show significant differences between the two irrigation regimes for each variety, according to the LSD (0.05) test; otherwise “ns” indicates non-significant. Means with different capital alphabetical letters show the significant differences between the two varieties according to the LSD (0.05).

The analysis of variance results for all measurements are presented in supplementary Table S1. The main effect of irrigation was only significant for S_s_ (P ≤ 0.01). When pooled over the two varieties, S_s_, S_p_, M_p_, M_s_, and SF_p_ values were 21.3%, 14.0%, 8.1%, 7.6%, and 16.8% higher in the SDI treatment than in the CK plots, respectively (Fig. 1; supplementary Fig. S1; all of the abbreviations and their corresponding explanations are provided in supplementary Table S2). SDI slightly increased the seed yield by 7.8% compared with the CK. Variety InVigor5440 had a numerically higher seed yield than variety InVigorL140P, but this difference was not significant.

#### Electrical measurements and root morphological traits

The root C in the SDI plot was 22.8% larger than of the CK when averaged across the two varieties (Fig. 1). Conversely, the root R and Z values in the SDI plot were 19.8% and 20.1% lower, compared with the CK. The root A, V and the dry weight per plant in the SDI treatment increased significantly by 22.7%, 30.9%, and 30.1%, respectively, compared with the CK when averaged across the two varieties (supplementary Fig. S2). The root L was significantly higher in variety InVigor5440 than in variety InVigorL140P. The root A, V and dry weight per plant were also numerically higher in variety InVigor5440 than in variety InVigorL140P; however, this difference was not significant. A similar result was observed under greenhouse conditions (data not shown).

#### Root cone diameter (D), model τD^3^, number of root branches and their relationships with anchorage strength

The root cone D and τD^3^ were significantly higher under root classification of 0.5 mm than for the 1 mm, regardless of irrigation regime or variety (supplementary Fig. S3). Generally, the soil cone D and τD^3^ were numerically higher in the SDI regime than in the CK plots, regardless of variety. However, this difference was not significant. The number of root branches per plant was 19.9% greater in variety InVigor5440 than in variety InVigorL140P.

#### Relationships of electrical measurements with root morphological traits

**Figure 2:**
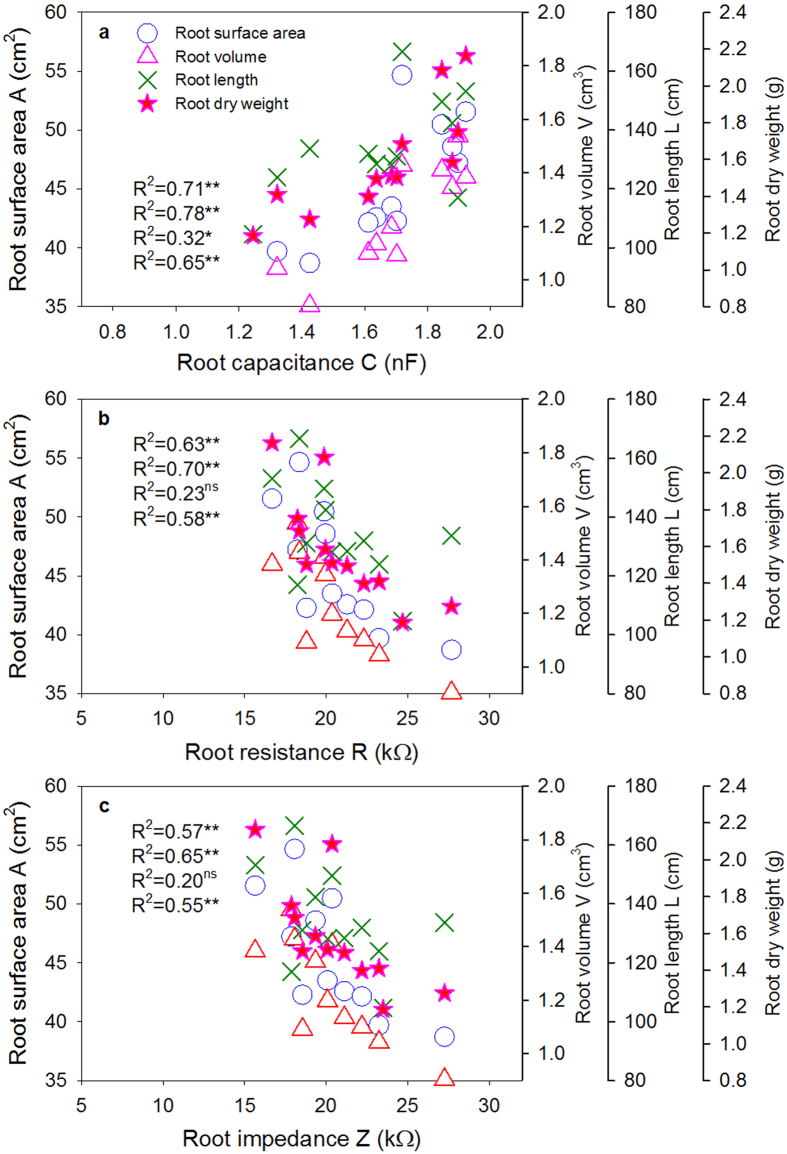
Relationships of (**a**) root capacitance, (**b**) root resistance and (**c**) root impedance with root morphological traits including root length, surface, volume and root dry weight per plant in Exp. I. **Indicates significant at p ≤ 0.01; *Indicates significant at p ≤ 0.05; ns indicates non-significant.

A regression analysis (Fig. 2) indicated that root C had positive relationship with the root morphological traits. However, the root R and Z displayed a negative relationship with these traits, especially in terms of the root A, V and dry weight per plant. All of these relationships were significant by a linear function model (P ≤ 0.05), except the relationships of root L per plant with root R and Z were not significant. Generally, the root C showed an even stronger relationship with these morphological traits than the root R and Z.

### Experiment II

#### Mechanical measurements and seed yield

**Figure 3:**
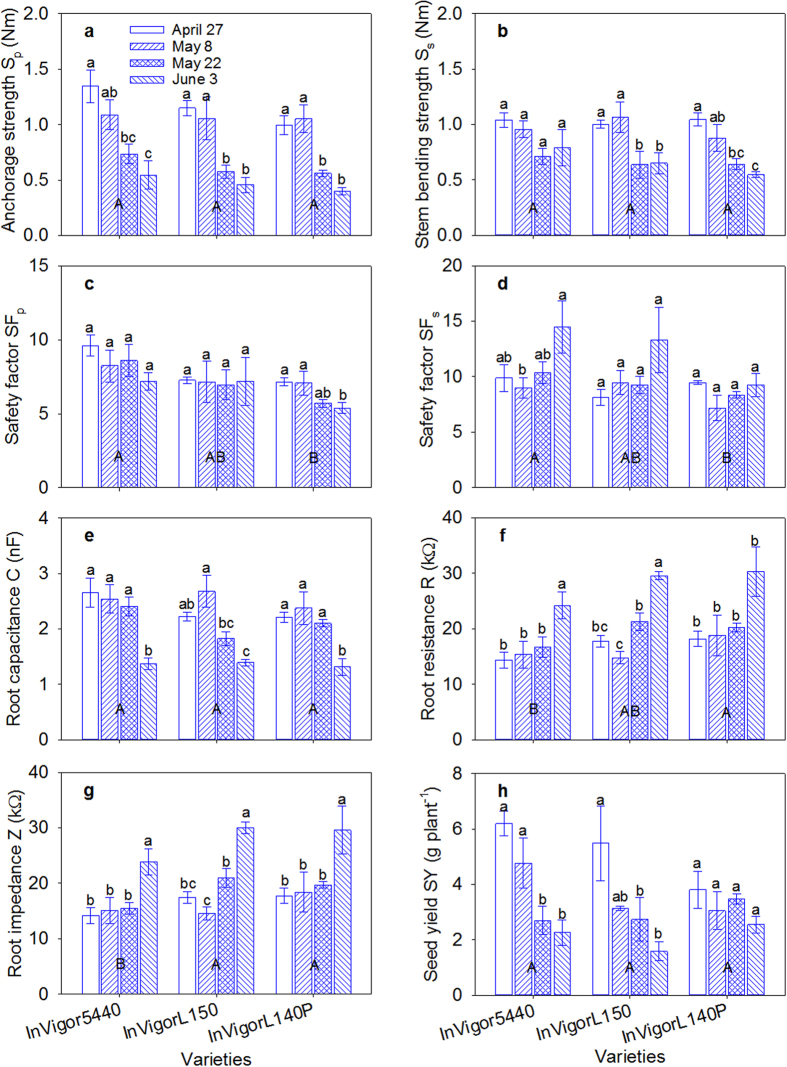
Effect of planting date on (**a**) anchorage strength, (**b**) stem bending strength, (**c**) root safety factor SF_p_, (**d**) stem safety factor SF_s_, (**e**) root capacitance, (**f**) root resistance, (**g**) root impedance and (**h**) seed yield among the three varieties of canola. Vertical bars above mean values indicate standard error of three replications. Means with different small alphabetical letters show significant differences between the planting dates for each variety, according to the LSD (0.05) test. Means with different capital alphabetical letters show significant differences between the varieties.

The planting date significantly affected the H, h_p_, h_s_, stem diameter, FreW, DryW, SM, BS, M_p_, M_s_, S_p_, S_s_, and SF_p_, which were significantly higher for the earliest planting date relative to the mid and late planting dates, except for BS and SF_s_, which were both lower for the earlier planting date but not significant, regardless of variety. S_s_, SF_p_, SF_s_, SM and FreW were numerically higher for the InVigor5440 variety than for the InVigorL140P and InVigorL150 varieties. However, this difference was not always significant (Fig. 3; supplementary Fig. S4). Seed yield was significantly greater for the earliest planting date than for the latest planting date. The InVigor5440 variety also showed the greatest seed yield among the three varieties, regardless of planting date.

#### Electrical measurements

Planting date had a significant effect on root C, R, and Z. Generally, root C gradually decreased as the planting date was delayed, and the root R and Z gradually increased (Fig. 3). The InVigor5440 variety generally had greater root C and lower root R and Z than the InVigorL150 variety, followed by the InVigorL140P variety.

### Experiment III

#### Mechanical measurements and seed yield

**Figure 4:**
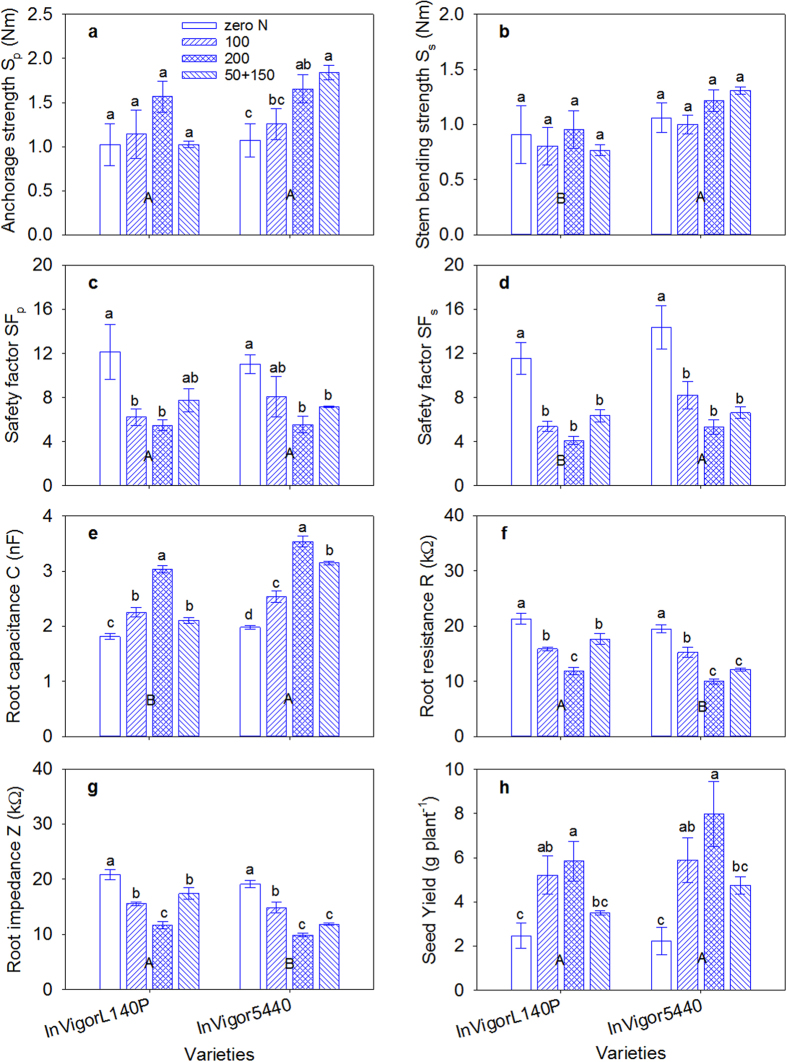
Effect of N fertilizer management on (**a**) anchorage strength, (**b**) stem bending strength, (**c**) root safety factor SF_p_, (**d**) stem safety factor SF_s_, (**e**) root capacitance, (**f**) root resistance, (**g**) root impedance and (**h**) seed yield between the two varieties of canola. Vertical bars above mean values indicate standard error of three replications. Means with different small alphabetical letters show the significant differences between four N fertilizer managements for each variety according to the LSD (0.05). Means with different capital alphabetical letters show the significant differences between two varieties according to the LSD (0.05).

The N fertilizer treatments significantly affected the plant H, h_p_, h_s_, stem diameter, FreW, SM, BS, M_s_, S_p_, SF_p_ and SF_s_ (supplementary Table S1). The plant H, stem diameter, FreW, SM, M_p_, M_s_ and S_p_ significantly increased as the N application rate increased, and the BS, SF_p_, and SF_s_ significantly decreased as the N application rate increased (Fig. 4; supplementary Fig. S5). The split application of N fertilizer (N_50+150_) significantly increased SF_p_ and SF_s_, relative to N_200_ treatment of the same N application rate, accompanying with significantly increase in BS, and significantly decrease in stem diameter, SM, M_p_, and M_s_. S_s_, SF_s_, SM, FreW and DryW were significantly higher for the InVigor5440 variety than for the InVigorL140P when averaged across all N treatments. Seed yield was highest under N_200_, followed by N_100_, N_50+150_ and zero N. InVigor5440 variety also showed higher seed yields when compared with the InVigorL140P variety.

#### Electrical measurements

The N fertilizer treatments, crop variety and their interactions significantly affected the root C, R and Z (supplementary Table S1). With increasing N application rate, the root C significantly increased and the root R and Z significantly decreased. The split application of N fertilizer (N_50+150_) significantly decreased the root C and increased the root R and Z, compared with the N_200_ treatment with the same N application rate, regardless of the variety (Fig. 4). InVigor5440 variety generally showed significantly higher root C and significantly lower root R and Z than the InVigorL140P variety.

#### Relationships among the mechanical properties and their associations with electrical measurements and seed yield

S_p_, S_s_, SF_p_, and SF_s_ were four important indicators that were used to evaluate the risk of stem lodging and root lodging. It was observed that the stem diameter, FreW, DryW, SM, BS, M_p_, and M_s_ were closely and positively correlated with S_p_ and S_s_ in all three field experiments (supplementary Fig. S6). However, these correlations with SF_p_ and SF_s_ were not strong, except for the negative correlations of SF_p_ and SF_s_ with M_p_ and M_s_. BS was always negatively correlated with S_p_ and S_s_ and positively correlated with SF_p_ and SF_s_.

**Figure 5:**
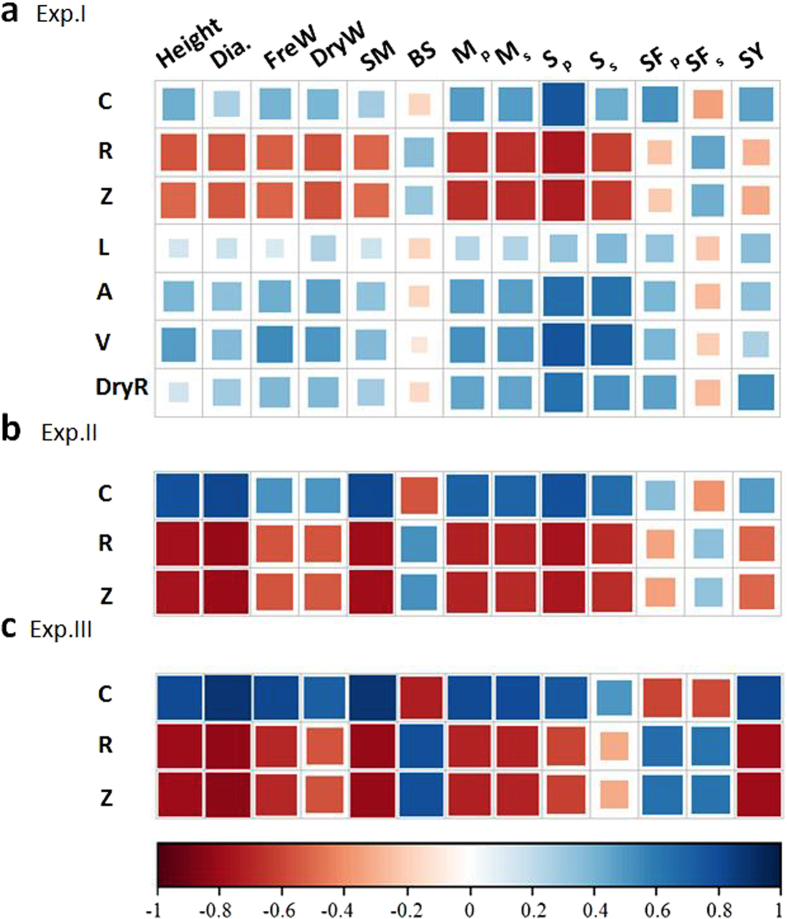
Colour map for the relationship of root capacitance (C), resistance (R), impedance (Z), root length (L), root surface (A), root volume (V), and root dry weight per plant (DryW) with plant height (H), basal stem diameter (Dia.), fresh weight per length (FreW), dry weigh per length (DryW), section modulus (SM), bending stress (BS), self–weight moment for root (M_p_), self–weight moment for stem (M_s_) and seed yield (SY). High colour density and larger square area indicates strong relationship. Blue and red colour represents positive and negative relationship, respectively.

**Figure 6:**
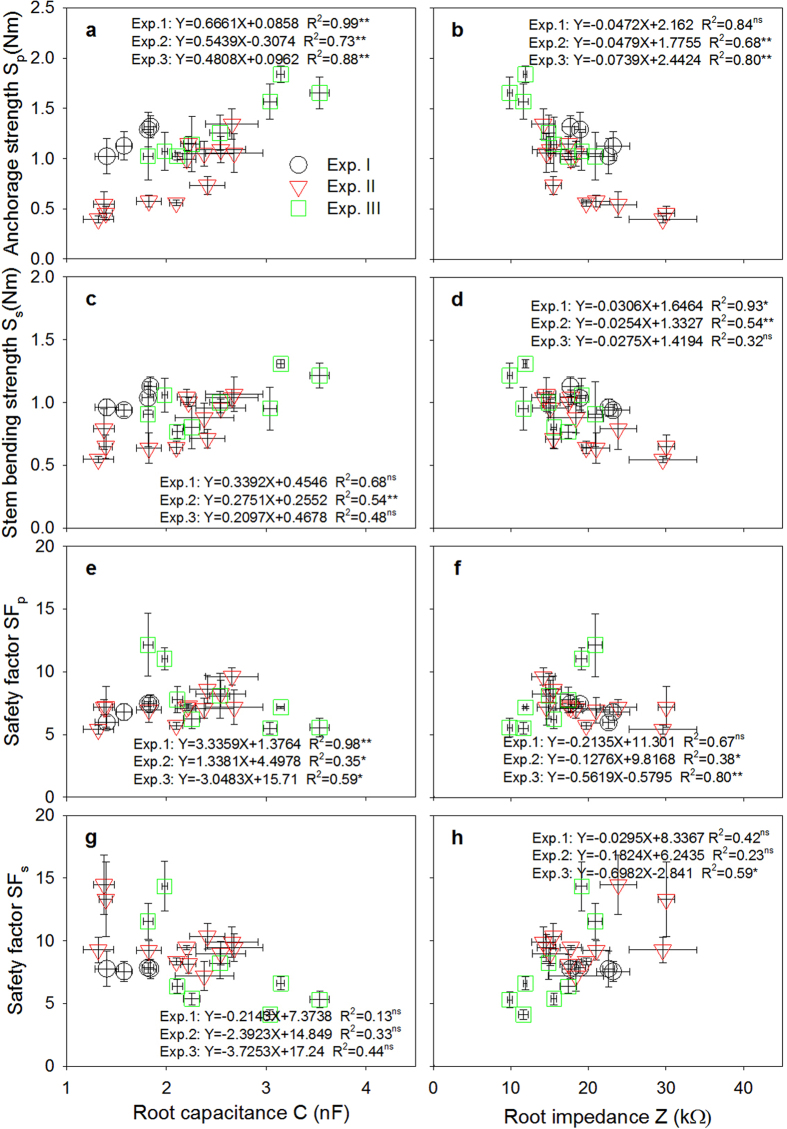
Relationship of root capacitance and root impedance with (**a,b**) root anchorage strength, (**c,d**) stem bending strength, (**e,f**) root safety factor SF_p_ and (**g,h**) stem safety factor SF_s_ in three field experiments. **Indicates significant at p ≤ 0.01; *Indicates significant at p ≤ 0.05; ns indicates non-significant.

**Figure 7:**
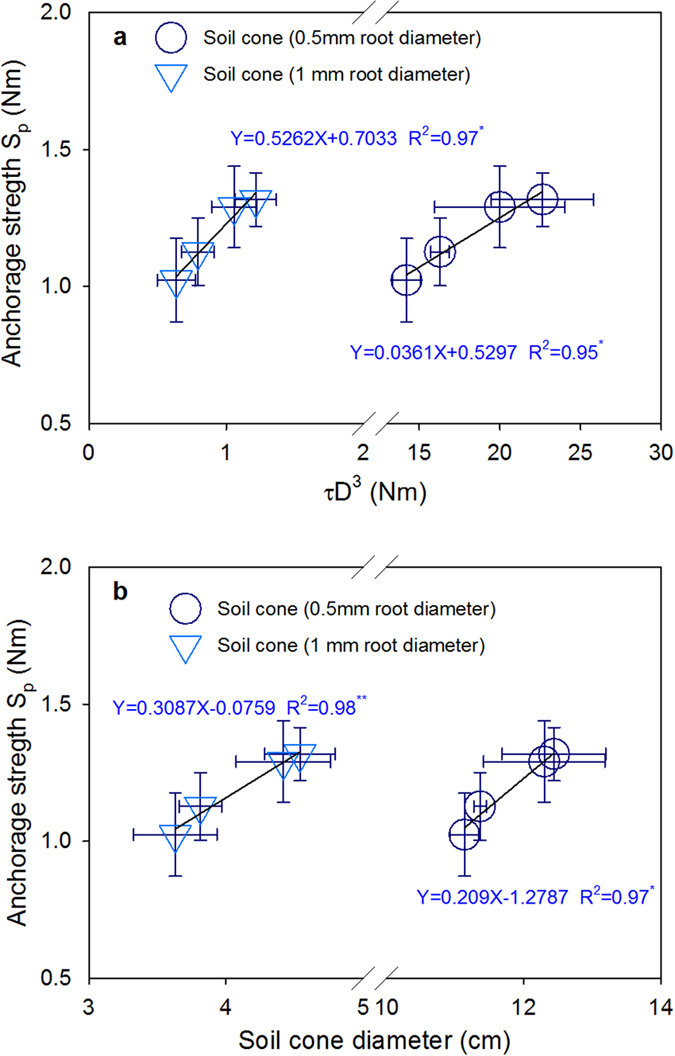
Relationships of anchorage strength with (**a**) model τD^3^ and (**b**) soil cone diameter. Root–soil cone diameter with 0.5 mm and 1 mm root diameter were estimated by root analysis system as described in Fig. 2. **Indicates significant at p ≤ 0.01; *indicates significant at p ≤ 0.05; ns indicates non-significant.

**Figure 8:**
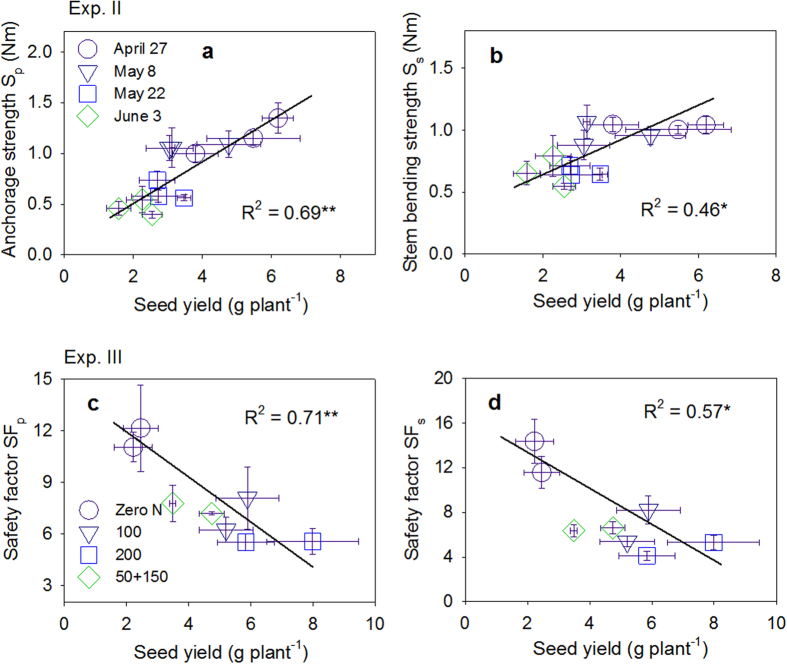
Relationships of canola seed yield with (**a**) anchorage strength, (**b**) stem bending strength, (**c**) root safety factor SF_p_ and (**d**) stem safety factor SF_s_. The data of Fig. a–b is from Exp. II and the data of Fig. c–d is from Exp. III. **Indicates significant at p ≤ 0.01; *Indicates significant at p ≤ 0.05; ns indicates non-significant.

The root C was always positively correlated with the lodging–related traits S_p_ and S_s_, and the root R and Z were always negatively correlated with S_p_ and S_s_, except for BS, among the three field experiments (Fig. 5). The relationships of S_p_ with root C, R, and Z were stronger than the other lodging–related traits and SF (Figs 5 and 6; supplementary Fig. S7). Several root morphological parameters, including root A, V, and the dry weight per plant, were also significantly correlated with S_p_ in Exp I (Fig. 5). The linear function model between the electrical measurements and the four most important indicators of lodging resistance (S_p_, S_s_, SF_p_, and SF_s_) are highlighted in Fig. 6 and supplementary Fig. S7. S_p_ showed strong relationship with *τ*D^3^ (Fig. 7a) and the soil cone D (Fig. 7b) (R^2^ = 0.95–0.98, P ≤ 0.05). Using a root diameter of 1 mm as a threshold value was more appropriate for predicting S_p_ than using a root diameter of 0.5 mm, which resulted in greater overestimation for soil cones D and *τ*D^3^ (Fig. 7). Figure 8 indicates that the seed yield had positive relationship with S_p_ but negative relationship with SF_p_.

## Discussion

To the best of our knowledge, no studies have identified simple and substitutable non–invasive indicators for assessing lodging risks in rapeseed canola plants. This study was the first to determine the feasibility of conducting electrical measurements in the field as a rapid and non–destructive method for indirectly assessing root S_p_. The theoretical explanation for the relationships between the electrical measurements and S_p_ are explored in Appendix I (supplementary material). The quantitative experimental results from the three field experiments further supported these strong relationships (Fig. 5). Among the considered electrical parameters, root C provided the best correlation with S_p_ in all field experiments.

In this study, two electrodes connected by an impedance LCR bridge meter, one at the plant stem base and the other in the soil medium, were used to obtain electrical measurements that were directly correlated with root morphological traits (Fig. 2). The theoretical concepts for root C and Z, as reliable *in situ* assessments of root size are partly summarized in Appendix I. The root–soil interface system had a C that was proportional to the charge accumulated on the membrane surfaces, which was positively related to root size (Fig. 2a). In addition, an electrical Z value was obtained that basically represents the resistance against the alternating current that is produced when a current passes from the soil to a root through a specific electrically conducting absorptive area on the root surface. Thus, the root Z measured in root–soil systems was negatively related with root size (Fig. 2b,c).

The device used to obtain electrical measurements in this study is less expensive and simpler, than the devices required for destructive lodging tests. Furthermore, it is more convenient to conduct electrical measurements under field conditions, and each measurement can be shown directly and digitally within a few seconds without requiring any comprehensive biomechanical calculations. Thus, this non–invasive method is highly recommended as an indirect method for assessing the lodging susceptibility among different genotypes or under different agronomic management systems when hundreds of samples are involved.

Notably, root C was closely related to seed yield in all field experiments. This relationship can be explained by the positive and consistent relationships of root C with several root morphological parameters in terms of root L, A and V, which have a significant impact on a plant’s potential capacity for nutrient absorption and water uptake^30^,^31^,^32^,^33^,^34^,^35^. Thus, plants with higher root C values generally have larger root systems that provide better access to soil water and nutrients than plants with smaller root C values, even in arid environments. Thus, these larger root systems could support great seed yields (Fig. 5). Several recent studies^36^,^37^ confirmed that using root C as a breeding criterion was efficient. In addition, these authors succeeded in using root C to select for high grain yields in wheat and barley.

Unlike the resistance to other biotic stresses, such as diseases or pests, the resistance to lodging is similar to the grain yield potential or yield performance, which are both characteristics of plant population rather than single plants and significantly interact with environmental factors. One negative aspect implies that one critical selection criterion for breeding lodging–resistance genotypes is difficult to identify from various lodging–related indices due to the complexity and interaction effect. However, selection according to plant characteristics, such as stem diameter or strength, that are associated with lodging resistance have been applied in breeding programs by some researchers^5^,^12^. On the positive aspect, our results imply that lodging tolerance may be paralleled with seed yield or yield potential (Fig. 8a,b; supplementary Fig. S6). Good lodging resistance allows plants to benefit from high levels of soil fertility and favorable environments and, consequently, approach their yield potential^7^. A study conducted by Ookawa *et al*.^38^ suggested that improved lodging tolerance in rice plants was an obvious feature linked to the superior grain yield of near–isogenic lines carrying the gene SCM2 due to the pleiotropic effects of the gene. Thus, it is possible to use one critical criterion, such as root size represented by root C, to improve crop lodging resistance while promoting seed yield in breeding programs or implementing agronomic management options.

By comparing SF_s_ with SF_p_ values among different treatments, this study allowed us to assess the likelihood of lodging risk caused by the stems and roots: like wheat^39^, teff^31^, and rice^30^, canola was identified to be more prone to anchorage failure than stem buckling. This was evidenced by the significantly lower SF_p_ than the SF_s_ values in Exp. I and Exp. II. The values of SF_p_ and SF_s_ were similar in response to N application (Exp. III). However, it is noted that the stem failure was only measured at 10 cm above the soil surface. Although the strength of the plant’s base was the most critical point for stem buckling in rice and wheat crops^5^,^8^, stem base may not be the weakest point in canola or other species, in which the stem tapers gradually from the base upwards. One study on barley suggested that the peduncle, rather than the base section, was regarded as the weakest point along the shoot^40^.

**Figure 9:**
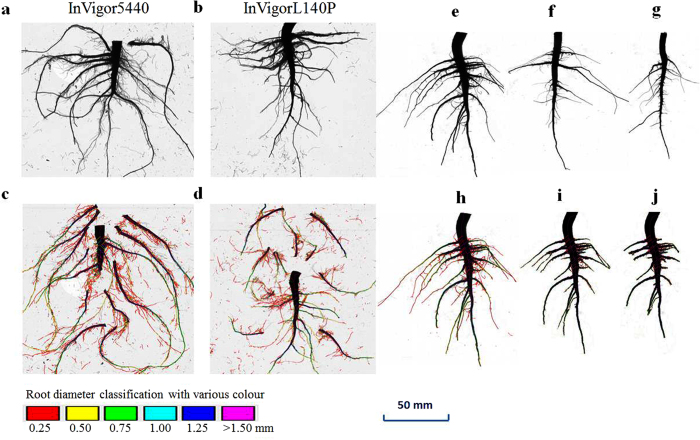
Diagram showing root analysis for root length, surface, volume and root–soil cone diameter calculation. (**a**,**b**) represent the intact root system for Variety InVigor5440 and InVigorL140P, respectively. To avoid the overlap of root systems, each lateral roots is separated carefully and emerged into the water for scanning and analysis with root diameter classification (**c–d**). The color of the skeleton drawn over the root is related to its three–dimensional diameter (as scale shown) and can be clearly identified. (**e,f,g**) represent intact root system of three different planting data, i.e. April 27, May 22 and June 3. Based on the original root system analysis (**h**) with colour root diameter classification, root–soil cone diameter within 0.5 mm and 1 mm root diameter can be estimated indirectly, as shown in (**i–j**), respectively.

In support to our hypothesis, this study showed that agronomic management options, significantly influenced the plant morphology and stability against lodging, including stem and root lodging. Variety InVigor5440 had larger root size than the other two varieties under the field conditions (Fig. 9a,b), and a similar result was observed under greenhouse conditions (data not shown). These results support our hypothesis that the lodging resistance variety such as InVigor5440 displayed greater stem diameter, SM, and DryW, compared with the other two lodging susceptible varieties (Figs 1, 3 and 4; supplementary Figs S1, S4, S5). Data analysis further showed that S_p_ and S_s_ were significantly correlated with several lodging–related parameters, including plant height, stem diameter, DryW, and SM among the three field experiments, which was similar to those in cereal crops reported in other studies^5^,^15^,^41^.

The plant morphological and mechanical parameters were not much different between the two irrigation regimes shown in Exp. I because serious drought stress did not occur during the growing season, as shown by the soil water content data. The total rainfall during the growing season was 214 mm (supplementary Fig. S8). Therefore, there was no yield penalty for the rain–fed crop (control treatment) relative to the SDI plots. However, it should be pointed out that there was a clear trend that the SDI treatment increased S_p_, SF_p_ and seed yield, regardless of the variety, although not significant. Further study is warrantied to verify the possible merits of SDI in terms of yield performance and lodging susceptibility under more drought–prone environments.

Canola plant growth can be directly suppressed by elevated temperature and drought events caused by delaying the planting date^17^,^20^. Site-specific optimum seeding date is suggested to be a successful strategy to avoid early summer heat and drought stresses and lead to greater canola seed yields^20^. In this study, delaying the planting date significantly increased the daily temperature during the flowering stage, which was the most critical period for causing pollen abortion^42^. For example, during the flowering stage, the number of days that the daily maximum temperature reached 30 °C was 0 for the earliest planting date vs. 5 for the last planting date (supplementary Fig. S8). Previously, high–temperature stress and possible drought were shown to seriously impede stem development and lignification and the translocation of carbohydrates to the root^42^. Thus, in this study, a thinner shoot diameter with smaller base dimensions and a diminishing tap root system were found for the intermediate and last planting dates compared with the earlier planting date (Fig. 9e–g), which resulted in weak S_p_ in Exp. II (Fig. 3).

**Figure 10:**
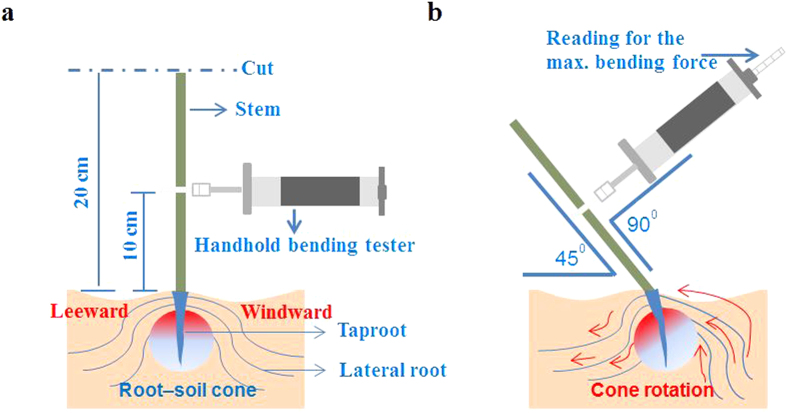
Measurements of anchorage strength using a modified prostrate tester and the movements of root systems during an artificial lodging test. The gradient colored circle indicates the root–soil cone, which is pushed out and rotates with root movement. A prostrate tester is attached to an adjustable mounted plate and placed perpendicularly at the middle of the residual basal stem.

Generally, tap roots grow deep into the soil and produce several secondary roots with strong breaking resistances to provide anchoring^6^ (Fig. 9e). This study indicated that the root systems of the canola plants from the late planting dates generally had few or no lateral roots that can resist a certain breaking force, because the diameter of laterals are generally less than 0.7 mm (Fig. 9e–g). The smaller lateral roots had lower bending strengths and did not contribute greatly to the S_p_, because it is well below the rotational root-soil cones (Fig. 10) of the plant stands^6^. Conversely, for the early planting date, the larger taproot systems were always accompanied by several large lateral roots, known as secondary branch roots, which emerged from the base of the stem and pointed radially outwards and tapered downwards (Fig. 9). The lateral roots with large radii and larger angular spread away from the vertical near the onset of the stem base and further branched, resulting in many tertiary roots; these roots formed an inverted crown shape, as described in another study for rice and wheat^30^.

Therefore, we speculate that the simple tap root structure without large lateral roots attached that resulted from the late planting date were not well anchored, which most likely reduced the S_p_. In addition, fewer or smaller lateral roots occupied a smaller volume of soil, leading to a small, circular root–soil cone that is prone to shearing when a certain rotational force is applied. Conversely, if a taproot system is well anchored by multiple strong lateral roots with great angular spread away from the vertical, such as those under early planting date, a greater elliptical root–soil cone volume could result in more resisting rotational forces^8^,^42^,^43^.

Increasing fertilizer N input from 50 to 200 kg N ha^–1^ in this study significantly increased the seed yield (Fig. 4h). However, this led to higher lodging risks with decreased SF_p_ and SF_s_ values (Fig. 4c,d). This paradoxical phenomenon was also observed in other studies^5^,^41^. The converse trend between seed yield and SF in responding to N application (as indicated by their negative relationship in Fig. 8c,d), implied that lodging could be a major constraint for yield performance under high–yielding conditions. Lower SF under higher N application was possible because higher N rates led to significant increases in M_p_ and M_s_ and decreases in BS. Higher N application rates also increased the S_p_ and S_s_, but not as strongly as the M_p_ and M_s_ (Fig. 4a,b; supplementary Fig. S5). High N application rates decreased the lignin deposition, vascular bundle area, and structural carbohydrate contents, which resulted in poor stem and root stiffness in *japonica* rice^16^. Previously, Pinthus^5^ indicated that higher N application rates dramatically reduced S_p_ and therefore resulted in a lower SF_p_ in wheat plants. More importantly, this study indicated that the N–split sidedressing practice (N_50+150_) increased the lodging resistance with higher SF_p_ and SF_s_ when compared with the N_200_ treatment with the same N application rate. Additional research regarding balanced N fertilizer management should be conducted to determine more powerful strategies for minimizing lodging risks while maintaining relatively high seed yields.

The root S_p_ can be calculated theoretically^32^ using the model *KτD*^*3*^ which was also illustrated in this study (Fig. 7; supplementary Fig. S3a–d). The dimensionless constant *K* (equation 5) value of 0.52 in the current study (Fig. 7; Y = 0.526X + 0.703; R^2^ = 0.97^*^), a slope coefficient that links the shear strength and root–soil cone diameter with S_p_, was within the range of 0.39 to 0.58, reported for cereal species, such as teff, wheat and barley^21^,^40^,^31^. Most of these previous studies forced the linear relationship between S_p_ and *τ*D^3^ to pass through the origin. However, Delden *et al*.^31^ suggested that this method was inconsistent with reality. They argued that an intercept of zero would imply that no anchoring force existed in the absence of a root plate; however, in fact, the shoot at the bottom of the plant base can lead to anchoring force.

These scholars also suggested that species with larger diameters generally have higher intercepts, such as sunflower, which had an intercept of 3.9 (Nm) from reanalyzing the data of another study^15^. In our study, an intercept value of 0.70 was obtained, which falls within the ranges described. Thus, this appears to indicate that both the root cone D and plant base diameter can significantly contribute to the root S_p_^31^. Consequently, equation (7) could be revised by including an intercept. However, different methods of measuring root cone D (such as careful excavation of the entire root–soil system^32^, by analyzing the structure of washed roots^33^, or by analyzing the root skeleton and classifying the roots by diameter, as presented in this study) can also cause certain variations.

The SF does not consider the forces generated by the wind, rain, storms, or additional weight of wet foliage or the effects of the interactions of the foliage with adjacent plants that may provide a degree of protection against lodging^31^,^22^. Notably, because canola has a spreading and elongated raceme rather than a straight spike, the classical lodging model developed for wheat^39^ and rice^30^ may not be valid for canola without modification or adopting other sophisticated models^40^. It is admitted that this method has shortcoming, as mentioned above, relative to other sophisticated models^21^,^22^,^30^ because the importance of wind loading and its related aerodynamics are omitted. Regardless of these shortcomings, this model still provides an essential method for comparing the mechanical abilities of a plant to withstand physical damage, as suggested by multiple scholars^11^,^30^,^31^.

## Conclusions

Our *in situ* field experiments indicate that the application of electrical measurements, especially for root C, can serve as a good, non–invasive indicator of root S_p_. Considering the simplicity and speed of electrical measurements without damaging the plant, these measurements can be applied to assess the lodging resistances among various agronomic practices and can be applied in breeding programs for selecting genotypes with both strong S_p_ and high yield potential.

Furthermore, it suggests that enhancing the root lodging resistance (increasing S_p_ and SF_p_) is more important than enhancing the stem lodging resistance (increasing S_s_ and SF_s_). Management options significantly influenced plant stability and seed yield. To minimize the risks of lodging while sustain high yield performance, farmers should adopt appropriate crop management practices, such as rational fertilizer management and optimum planting date, and select varieties with strong S_p_ represented with high root C reading.

## Materials and Methods

### Experimental design and field management

Three field experiments were conducted in 2015 on the Central Experimental Farm of Agriculture and Agri–Food Canada in Ottawa, ON, Canada (45°23′ N, 75°43′ W). The soil chemical properties are shown in supplementary Table S3. Weather data were collected using an automatic weather station near the experimental fields. The daily maximum temperature, minimum temperature, maximum wind speed and rainfall during the growing season are presented in supplementary Fig. S8.

### Experiment I_subsurface drip irrigation trial

Experiment I was a randomized complete block design in a split-plot arrangement, consisting of canola varieties subjected to two water regimes. The whole plots consisted of two irrigation patterns (subsurface drip irrigation, SDI; without irrigation as a control, CK). Two varieties, InVigorL140P and InVigor5440 were the subplots. All three field experiments had three replications. Based on our preliminary evaluation of many varieties in greenhouse experiments, variety InVigor5440 had a larger root size and stronger S_p_ values than the other varieties under most conditions.

Each subplot dimension was 8 m × 1.5 m and contained 8 rows of canola with row spacing of 17.8 cm. The plots were sown 6 May 2015 using a seeding rate of 5 kg ha^−1^ and a seeding depth of 1–2 cm. The experiment followed provincial recommended management practices for weed control^2^. Field preparation included chisel plowing to a depth of approximately 15–20 cm in the fall (using a DMI Elco Tiger machine) and cultivating to a depth of 10–15 cm in the spring (using a C–shank cultivator) before broadcasting N fertilizer (urea) at 100 kg N ha^−1^ before planting. All plots received adequate P (as superphosphate) and K (as potash) fertilizer during land preparation based on soil test recommendations, as described in supplementary Table S3.

To minimize seepage between different irrigation patterns, the main plots were separated by a conservation zone of 3 m to prevent the movement of water from the subsurface drip irrigation (SDI) system to the CK plots. To prepare the SDI plots, beds were established in the SDI area on 5 May 2015 using Triple K “S” tine cultivation. The SDI plots were supplied with irrigation water through a Senninger 15 PSI Pressure Regulator to reduce the water pressure from the city water supply valve. Water flowed to an irrigation tape system (Aqua Traxx Premium Drip), which was buried 12 cm under the surface with emitters spaced 30.5 cm apart, on the same day of seeding. The irrigation pipes were positioned in the same direction as the rows of canola. Two lengths of tape were placed in each SDI plot at a distance of 75 cm from each other. The flow of water into the SDI plot began at 09:00 each day and continued for 13 minutes at a flow rate of 2 mm d^−1^, by an electric water control console (Yardworks, Electronic Water Timer).

### Experiment II_planting date trial

Experiment II was a randomized complete block design in a split–plot arrangement, where the four planting dates (April 27, May 8, May 22, and June 3) were the whole plots, and the three varieties, InVigorL140P, InVigor5440 and InVigorL150 were the subplots. Each subplot was 8 m long and consisted of 16 rows of canola with a row spacing of 17.8 cm. In all plots, N fertilizer (urea) was split–applied at a rate of 50 kg N ha^−1^ before planting and sidedressed at a rate of 50 kg N ha^−1^ at the rosette stage. Other agronomic management practices and field preparation were the same, as described above in Exp. I.

### Experiment III_N fertilizer management trial

Experiment III was conducted to test the responses of two canola varieties, InVigorL140P and InVigor5440, to the timing (preplant vs. split application) and rate (0, 100, 200, and 50 + 150 kg N ha^−1^) of N fertilizer application. The experiment was a randomized complete block design. Each plot was 8 m long and consisted of 16 rows with a row spacing of 17.8 cm. The plots were sown on 4 May 2015. Other agronomic management practices and field preparation were the same, as described above in Exp. I.

## Sampling and Data Collection

### Lodging–related traits

The lodging–related traits were determined at one week before the physiological maturity (BBCH 89). Before sampling, eight uniformly–spaced canola plants from an inner row (row 4 from the border) were tagged, numbered and labeled in each plot. The crop height (H), defined as the distance between the soil surface and the top of the plants, was measured. The lodging simulation procedure was adopted from previous studies^12^,^30^ with some modifications, and the employed conceptual model was similar to that of Delden *et al*.^31^. The selected plants were cut off at a height of 20 cm. Simulated lodging was performed on the remaining stem base of each plant, by using a modified prostrate tester machine (DIK–7401, Daiki Rika Kogyo Co., Ltd, Saitama, Japan). The device was attached to an adjustable mounted plate at the middle of the residual basal stem (10 cm point) and the restoring anchorage moment supplied by its root system was recorded when the stem was pushed to an 45^o^ angle from the vertical position (Fig. 10). The rotation speed was approximately 2.0^o^ S^−1^.

After the S_p_ measurements, the plant without the basal stem was balanced on a thin, smooth metal tube, and the distances between their balance point and base end were recorded as the centers of gravity of the stem (h_s_). The basal stem was cut at the ground level, and was combined with its corresponding above-ground plant as a whole by using transparent tape, and then the centers of gravity of the entire plant (h_p_) was recorded as similar as h_s_. The fresh weight of these basal stem segments (FreW) and those of the other plants were recorded immediately to avoid any weight loss. The basal stem segments were packed in plastic bags and brought to the laboratory to determine their stem breaking resistance by using the three–point bending prostrate tester, as described by Delden *et al*.^31^ with some modifications. The distance between the fulcra of the tester was set at 15 cm. The center of the basal stem, where the breaking resistance was measured, was aligned horizontally with the middle point between the two fulcra. Then, the crosshead of the pushing probe was attached to the center point of the basal stem to bend the stem vertically at a rate of approximate 50 mm min^−1^ until it eventually buckled, which was identified by cracking noises. The diameters of the minor and major axes in an oval cross section for the basal stem segment near the lowest position were measured using a digital caliper (General ^®^). The dry weight of the basal stem segment was measured after oven drying at 80 °C until a constant weight was achieved to calculate the dry weight per unit length (DryW).

Although the maximum breaking strength (equal to the maximum self–weight moment, S_s_) and root S_p_ can be applied to identify the risks of crop lodging due to stem and root failure, respectively, the concept of “safety factor” (SF) was introduced two decades ago to identify lodging susceptibility^11^. The SF represents the number of times a support organ (such as stems and roots) can bear the self–weight moment (M) of the organ that it is supporting. The SF against stem buckling (stem lodging, SF_s_) and against anchorage failure (root lodging, SF_p_) are given as follows:


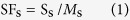



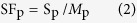


where S_s_ is the maximum self–weight moment (Nm) that the stem can support before it fails, which is calculated as S_S_ = *F*_max_ × *L* /4. Here, *F*_max_ is the maximum force (N) a stem will withstand before it fails and can be measured using a three–point bending test, as described above. L is the distance (cm) between the supporting points in the three–point bending test and was set to 15 cm. S_p_ is the root anchorage strength (Nm), which indicates the maximum moment at θ^o^ from the vertical stand that a root system can withstand before rotating further in the soil (see the measurement method described above).

The parameters M_s_ and M_p_ are the self–weight moments (Nm) at θ^o^ from the vertical stand for the stem and entire plant and are calculated as follows:


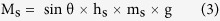



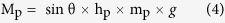


where θ is inclination angle from the vertical stand and h_s_ and h_p_ are the heights (cm) of the centers of gravity of the stem and entire plant, respectively. In addition, m_s_ and m_p_ are the fresh weight (g) of the stem (except for basal 10 cm segment) and entire plant, respectively, and g is the acceleration due to gravity (N kg^–1^).

The section modulus (SM, mm^3^) and bending stress (BS, N mm^–2^) are calculated as follows:


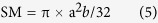



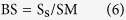


where “a” is the outer diameter of the minor axis in an oval cross–section and “b” is the outer diameter of the major axis in an oval cross–section.

S_p_ can also be estimated using another method. Crook and Ennos^32^ developed an equation for theoretically integrating the soil shear strength and root cone diameter, which had also been verified by other researchers^21^,^31^.


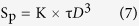


where τ is the soil shear strength (N m^–2^) that can be measured using a shear vane; D is the root cone diameter (cm); and K is a dimensionless constant.

### Electrical measurements

On the same day when lodging–related traits were recorded, a dual display LCR meter (Model 879B, B&K Precision Corporation, USA) was used to record the electrical C, Z and resistance (R) at a frequency of 1 kHz, following the procedure outlined in the instruction manual. To achieve optimal readings, standard calibration was performed before making any measurements. A ferric rod (6.6 mm diameter, 45 cm long) was inserted 150 mm into the field soil and 90 mm from the base of the canola stem. The negative electrode of the LCR meter was attached to the ferric rod that was inserted into the soil and the positive electrode was attached to a stainless needle with a diameter of 1 mm that was embedded into the stem (near the collar root and soil surface). The position of the stem electrode was chosen carefully because it significantly influenced the electrical measurements^26^. Each reading was taken after allowing the meter to stabilize for 6 s to obtain a constant reading.

### Root morphological traits

Fine roots were collected from Exp. I for further root morphological analysis. After finishing the electrical measurements and anchorage test in the field, the root systems of eight corresponding plants were carefully excavated by using a spade to extract their root balls, with a soil surface area of approximately 360 cm^2^ (18 × 20 cm) and to a depth of 30 cm. The root balls were taken to the laboratory and immersed in tap water for 1 hour. Next, the root balls were thoroughly washed using a self–made root clearance device to remove the field soil. Great care was taken during washing to minimize the loss of fine roots and to ensure that the root system remained mechanically intact (Fig. 9a–d). Fine roots were placed in plastic bags to prevent desiccation and stored in the fridge at −20 °C before further morphological examination.

The root systems were scanned to determine the root L, A, V and number of branches per plant by using a scanner (Epson Expression 1640XL, Epson America, Inc., USA) and a root image analyzer (Win RHIZO, Regent Instrument Inc, Quebec, Canada), as shown in Fig. 9a,b. To avoid any overlap of the root system and improve the data precision, each lateral root was separated from the tap root and emerged in water for further scanning and analysis (Fig. 9c,d). The root–soil plate diameter was estimated indirectly, based on the root skeleton drawn by the image analyzer using a root diameter class certification (Fig. 9h). The color of the skeleton drawn over the root was related to its three–dimensional diameter for clear identification (Fig. 9c,d and h). Only the lateral roots with diameters of more than 1 mm (Fig. 9j) or 0.5 mm (Fig. 9i) were considered for the root–soil plate diameter calculations. It was hypothesized that the root systems with diameters of more than 0.5–1 mm have lignified and have fixed structures that are not deformed during artificial lodging. Root diameters of 0.5 and 1 mm were selected as class certification standards because the snap or bending point when the roots were pulled over generally occurred at approximately 0.5–1 mm during the preliminary tests. Each individual root system was dried at 70 °C for 72 hours before weighing.

## Data Analysis

All the experimental data were first tested for normality with SAS procedure Univariate, prior to analyses of variance (ANOVA)^44^. No transformation was applied to any data because they were homogenous. In each experiment, the two-way ANOVA was conducted to determine treatment effects based on a randomized complete block design with split-plot arrangement (Exp. I and Exp. II) or on a randomized complete block design (Exp. III), using the MIXED procedure in SAS. Once a significant (P < 0.05) treatment effect was determined by the ANOVA, the treatment mean comparisons with the conservative letter grouping were made at the 95% level of confidence by the Least Significant Difference (LSD) method. Linear regressions between electrical measurements, lodging-related parameters and seed yield were performed using the REG procedure in SAS. The Pearson simple correlations between the measured parameters were evaluated using the CORR procedure, and the displayed maps were drawn using the R software^45^. All statistical analyses were performed at the 5% level of significance. All of the other figures were prepared using the SigmaPlot ver. 13.0.

## Additional Information

**How to cite this article**: Wu, W. and Ma, B.–L. A new method for assessing plant lodging and the impact of management options on lodging in canola crop production. *Sci. Rep.* **6**, 31890; doi: 10.1038/srep31890 (2016).

## Supplementary Material

Supplementary Information
