# Supplementary material for: A new method for assessing plant lodging and the impact of management options on lodging in canola crop production
Source: Sci Rep. 2016 Aug 24;6:31890. doi: 10.1038/srep31890 (PMC4995411; doi:10.1038/srep31890)
Supplement: Supplementary Information [file srep31890-s1.pdf]

A new method for assessing plant lodging and the impact of management options on lodging in canola crop production

Wei Wu[1,2] & Bao–Luo Ma[1,*]

[1]Ottawa Research and Development Centre, Agriculture and Agri–Food Canada, 960 Carling Ave, Ottawa, ON, K1A 0C6, Canada

[2]College of Agronomy, Northwest A&F University, Yangling 712100, Shaanxi, China

[*] To whom correspondence should be addressed. E–mail: Baoluo@agr.gc.ca (B. Ma). Tel.:+01 613–759–1521; Fax: +01 613–759–1701.

Appendix I:

Theoretical hypothesis regarding the relationships between the electrical measurements and anchorage strength (S_p)

Several crop lodging models were used to interrelate and assess the biomechanical properties that explain lodging, as mentioned in the Introduction section. According to the concepts used in their models, the lodging resistance of a plant depends on the following three main factors: (1) the extent of the forces that the plant is subjected to; (2) the maximum stem bending strength S_s for resisting buckling; and (3) the maximum root anchorage strength S_p for resisting overturning when an external force is applied[21,39].

Root lodging, caused by anchorage failure is considered more prevalent than stem lodging (see Introduction section and our experimental verification). Herein, we only consider S_p and determine its possible relationship with electrical measurements.

Anchorage failure involves bending of the roots at their base (please see the deformation of lateral and tap root systems shown in Fig. 10b relative to Fig. 10a) and axial movement of the leeward and windward roots through the root–soil plate rotation (the colored circle representing movement as a plant overturns in Fig. 10b relative to Fig. 10a). The total root restoring strengths from these motions provide a bending moment to resist root lodging. This root lodging anchorage model was proposed by Ennos[10] who hypothesized that the total S_p includes two separate components, the resistance of the root to bending and the resistance of the root to axially movement through the soil media.

$$S_p = M_b+M_a \qquad (1)$$

where S_p is the total anchorage strength (Nm); M_b is the root bending strength (Nm); and M_a is the root resistance to axial motion (Nm).

The experimental results of Ennos[10] further supported this anchorage model and suggested that the two components of the model are approximately equal in magnitude.

$M_a = M_b$ (2)

In Ennos's model, each individual root is considered as a standard and lignified cylinder that emerges from the base of the stem at a certain angle, ϕ, and a vertical angle of ƛ from the plane of the lodging force applied (supplementary Fig. S9). A distance of L_{perp} from the lodging plate will be projected, as shown in supplementary Fig. S9, and the perpendicular distance, L_{par}, will be calculated as $L\sqrt{(1 - sin^2\phi\ sin^2ƛ)}$ using the Pythagorean theorem. Lignified roots are stiff and resist bending. Thus, a perpendicular force, *F*, must be applied to the root tip to bend it to an angle of α. When the base of the stem was rotated by this angle, the root retained its orientation and bent at its base at an angle of β, which was equal to $\alpha\sqrt{(1 - sin^2\phi\ sin^2ƛ)}$. Then, the resistance force, *f*, at the tip of the root could be calculated as follows according to Hooke's law $F\sqrt{(1 - sin^2\phi\ sin^2ƛ)}$.

This resistance force was applied at a perpendicular distance of L_{par} from the axis of rotation. Thus, this bending moment can be calculated as $m_b = f\,L_{par}$. Then, the bending moment of each individual root can be calculated as follows:

$m_b = FL(1 - sin^2\phi\ sin^2ƛ)$ (3)

Because individual roots can be subjected to three–point bending tests, the maximum force *F* (N) that the root can withstand before falling can be estimated as follows:

$F = k \times BS \times SM$ (4)

where k is the reciprocal of the distance (mm) between the supports in the three–point bending test; BS is the bending stress (N mm^{-2}); and SM is the section modulus (mm^3). The root morphology can be recognized as a standard solid circle; thus, SM= $\pi d^3/32$, where d is the radius

of the root. Then, equation (3) can be written as follows:

$$m_b = k \times \pi \times BS \times d^3 \times L(1 - sin^2\phi\, sin^2\lambda)/32 \quad (5)$$

The total anchorage component, M_b, of the root bending moment is the sum of all of the individual roots.

$$M_b = \sum_{i=1}^{n} k\pi BS{d^3}_i L_i(1 - sin^2\phi_i\, sin^2\lambda_i)/32 \quad (6)$$

By combining equations (1) and (2), the total anchorage strength S_p can be estimated as follows:

$$S_p = \sum_{i=1}^{n} k\pi BS{d^3}_i L_i(1 - sin^2\phi_i\, sin^2\lambda_i)/16 \quad (7)$$

We hypothesize that BS is a function of the cell wall components, such as cellulose and lignin, and could be constant[13]. Therefore, if the roots are disturbed symmetrically in the soil at the base of the plant, S_p will mainly depend on the radius, length and spreading angle of an individual root under certain directions in which the lodging force is applied.

If we consider the root area (A, the macro–scale geometrical surface area defined by 2πdL) or root volume (V) as an independent variable, equation (7) becomes

$$S_p = \sum_{i=1}^{n} \frac{k\, BS\, A^3(1-sin^2\phi_i\, sin^2\lambda_i)}{128\pi^2 {L^2}_i} \quad (8)$$

$$\text{or } S_p = \sum_{i=1}^{n} k\, BS\, \sqrt{V_i/\pi L_i}\, V_i(1 - sin^2\phi_i\, sin^2\lambda_i)/16 \quad (9)$$

Dalton[28] developed a conceptual model for estimating root morphology in terms of the A, V and biomass by using the root C, which is measured by inserting one electrode at the base of the stem and one electrode in the rooting soil. This method is based on measuring the electrical C of an equivalent parallel resistance–capacitance circuit formed by the interface between the root and soil solution surface. Several researchers have observed strong correlations between root C and root biomass[24-26].

According to Dalton's model[28] root tissue segments resemble axially symmetric cylinders and

are considered as a standard and lignified cylindrical condenser with length, L, and dielectric constant, ε_i. Dalton's model indicated the presence of an inner cylindrical conductive condenser with an effective radius of r_{i1} for xylem section separated by root tissues whose outer surface was in contact with an exterior conductor (water or nutrient solution) at an effective radius of r_{i2}. In this case, C is given as

$$C_i = \frac{\varepsilon_i A}{4\pi \mathrm{In}\left[\frac{r_{i2}}{r_{i1}}\right] r_{i2}} \quad (10)$$

This equation shows that the root A for each root section is positively correlated with the root C. The total effective C is additive with respect to each root section, thus, equation (10) can be written as

$$C = \sum_{i=1}^{n} \frac{\varepsilon_i A_i}{4\pi \mathrm{In}\left[\frac{r_{i2}}{r_{i1}}\right] r_{i2}} \quad (11)$$

As r_{i1} boundlessly approaches r_{i2} where the separation d_i is equal to r_{i2} by subtracting r_{i1}, equation (11) can be revised as follows:

$$C = \sum_{i=1}^{n} \frac{\varepsilon_i A_i}{4\pi d_i} \quad (12)$$

If Dalton's and Ennos's models are combined, the relationship between S_p and root C becomes

$$S_p = \sum_{i=1}^{n} \frac{k\pi BS\, d^3{}_i C^3 (1 - sin^2\phi_i\, sin^2\lambda_i)}{2\varepsilon^3{}_i L^2{}_i} \quad (13)$$

Equation (13) shows that a positive relationship exists between S_p and root C. Except for root C, which has been employed to assess root morphology parameters, such as the root biomass, L, A, V and function, C measurements can be used to assess environmental stress conditions (such as hypoxia and anoxia) or abiotic stresses (such as drought and heat). Associated investigations of electrical measurement have also been conducted to evaluate freeze or cold injury[45].

Conversely, as shown for root C, root Z is basically the resistance between the alternating current

when the current passes from the root–soil system. The root Z is inversely proportional to its root C. A detailed theoretical description of root Z is presented in other study[29]. According to its theoretical model[29], root Z can be calculated as follows:

$$Z = \frac{\rho_{root}\, L_{mean}}{A} \xi\eta\zeta \qquad (14)$$

where ρ_{root} is the electrical resistivity of the root interface and is equal to the resistivity of the soil near the root interface; L_{mean} is the average length of the root segments; A is the surface area of the root segment; and the dimensionless coefficient ξ considers the mutual electric shielding effects of the roots. The dimensionless coefficient η represents the possible effects of mechanical root injury; the dimensionless coefficient ζ indicates a negative error when assessing the absorbing root surfaces due to other electric currents flowing through various pathways rather than through the measured root segment. These values are generally approximately 1[29]

If equation (14) is combined with equation (8), then S_p can be calculated as follows:

$$S_p = k\ BS\ \rho_{root}^3\ L\xi^3\eta^3\zeta^3 \frac{(1-sin^2\phi_i\, sin^2\lambda_i)}{128\pi^2 z^3} \qquad (15)$$

Although the models of Dalton, Ennos, Aubrecht *et al.*[10,28-29] were not developed for canola, these models' concept can be applied and analogized to canola plant according to those theoretical bases.

Goodman *et al.*[6] developed a simple model for the anchorage failure of oilseed rape. By contrast with wheat and rice, which have fibrous root systems, oilseed rape has a tap root system with few laterals roots. Thus, they suggested that the roots of oilseed rape plants were anchored mainly by a rigid tap root rather than by lateral roots. These authors suggested that two components exist for S_p, the resistance of the tap root to bending below the soil surface and the restoring force of the soil to lateral compression. Because laterals roots were ignored, the first components can be simplified as follows based on equation (5):

$M_b = k \times \pi \times BS \times d^3 \times L / 32$ (16)

where the tap root is considered as a rigid rod of length L and radius d.

The second component of anchorage failure is based on engineering theory and can be predicted as follows:

$M_a = 9\tau dL^2$ (17)

$S_p = M_a + M_b = k \times \pi \times BS \times d^3 \times L / 32 + 9\tau dL^2$ (18)

If A and V are treated as independent variables, equation (18) can be rewritten as equations (19) and (20), respectively.

$$Sp = \frac{k \times BS \times d \times A}{64} + \frac{9L\tau A}{2\pi} \quad (19)$$

$$Sp = \frac{k \times BS \times d \times V}{32} + \frac{9\tau V^2}{d^3\pi^2} \quad (20)$$

Thus, by combining equations (19) and (20) with equations (12) or (14), we can obtain equations similar to equations (13) and (15) and illustrate the relationship of S_p with root C and Z.

This section suggests that S_p is related to the root diameter, L, its geometrical construction (Equation 7), and therefore to root A and V (Equation 8, 9, 19 and 20), which can be represented with some indicator in terms of root C or Z (Equation 13 and 15). These relationships provide a theoretical basis for the relationship between S_p and the electrical measurements. Generally, S_p is positively correlated with electrical C and negatively correlated with Z, as shown in the equations presented above.

Furthermore, other variable parameters may exist (such as BS, d, L, ε_i, τ) that influence the relationships of S_p with electrical measurements. However, these traits behave in a conservative manner and in proportion to root size in a fairly uniform manner. Keeping this opinion in mind, these simple models partially link root C to root morphological parameters, such as root radius, length, A, V and S_p. It should be noted that the above hypothesis is based on the assumption of

constant constructional characteristics. For example, enlarging the angular spread of the lateral roots vertically could strengthen S_p without much investment in root biomass[5], which would not increase the L, A, V and corresponding root C but would allow most of these traits to remain constant (except for Goodman's model excluding lateral roots' contribution to S_p). The actual behaviors of their relationships remain to be shown experimentally under field conditions.

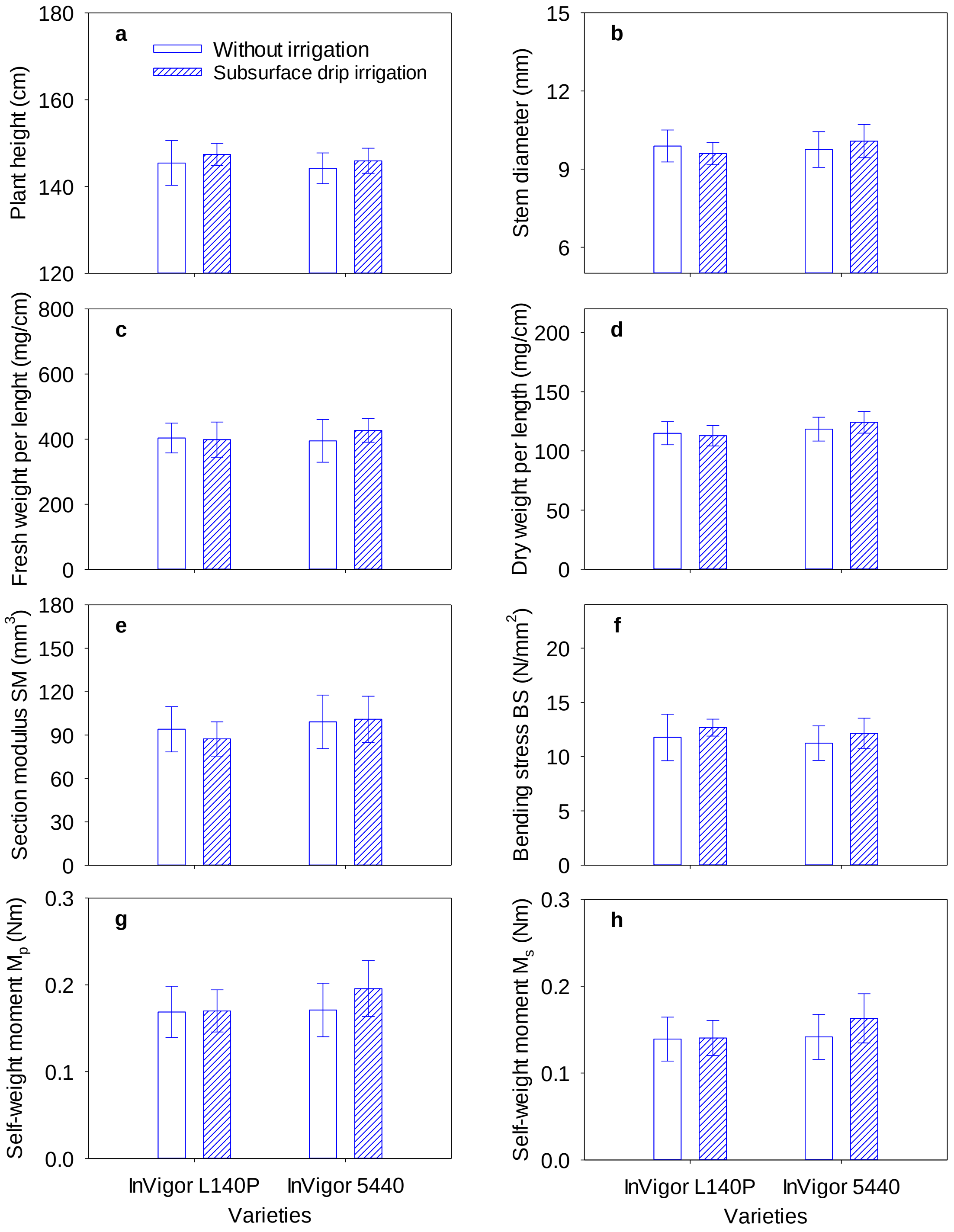

Fig. S1. Effect of irrigation regime on plant height (a), stem diameter (b), fresh weight per length (c), dry weight per length (d), section modulus (e), bending stress (f), self–weight moment M_p (g) and self–weight moment M_s (h) between two varieties of canola in 2015. Vertical bars above mean values indicate standard error of three replications. Significant differences between two irrigation regimes or between two varieties were not found according to LSD (0.05).

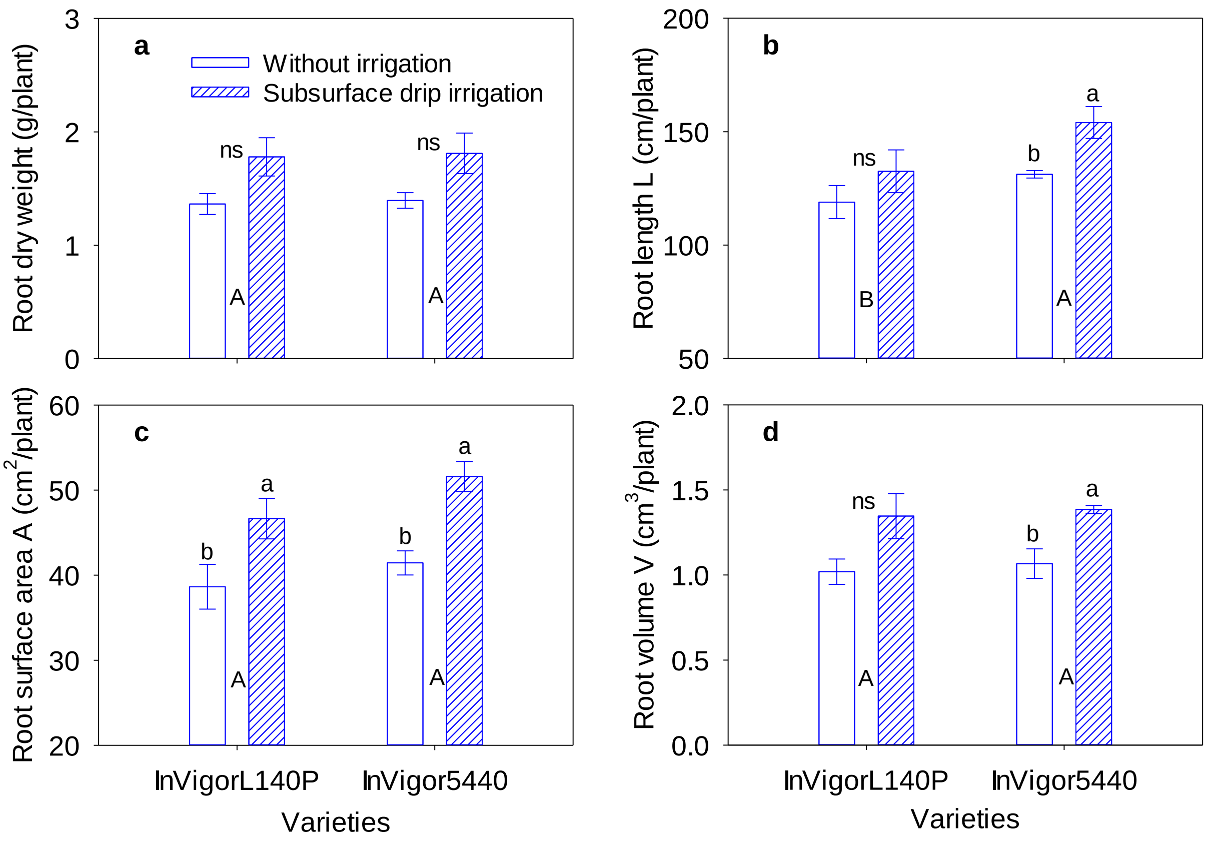

Fig. S2. Effect of irrigation regime on root dry weight (a), root length (b), root surface area (c), and root volume per plant (d) between two varieties of canola in 2015. Vertical bars above mean values indicate standard error of three replications. Means with different small alphabetical letters show the significant differences between two irrigation regimes for each variety according to LSD (0.05); otherwise "ns" indicates non-significant. Means with different capital alphabetical letters show the significant differences between two varieties according to LSD (0.05).

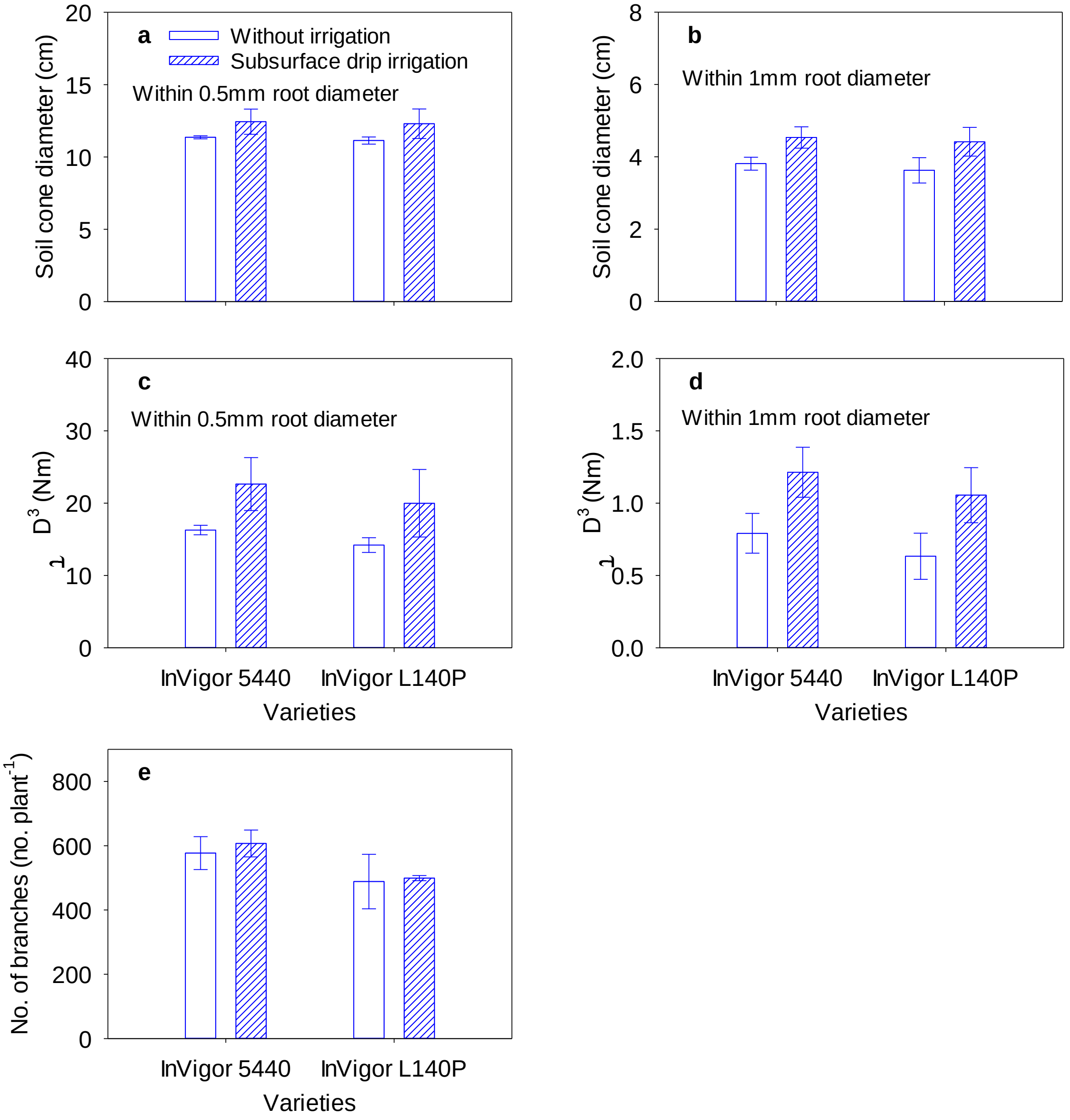

Fig.S3. Effect of irrigation regime on soil cone diameter (a and b), τD^3 (c and d), and number of branches per plant between two varieties of canola in 2015. Soil cone diameter and τD^3 are calculated based on 0.5 mm root diameter classification (a and c) and 1mm root diameter classification (b and d). Vertical bars above mean values indicate standard error of three replications. Significant differences between two irrigation regimes or between two varieties were not found according to LSD (0.05).

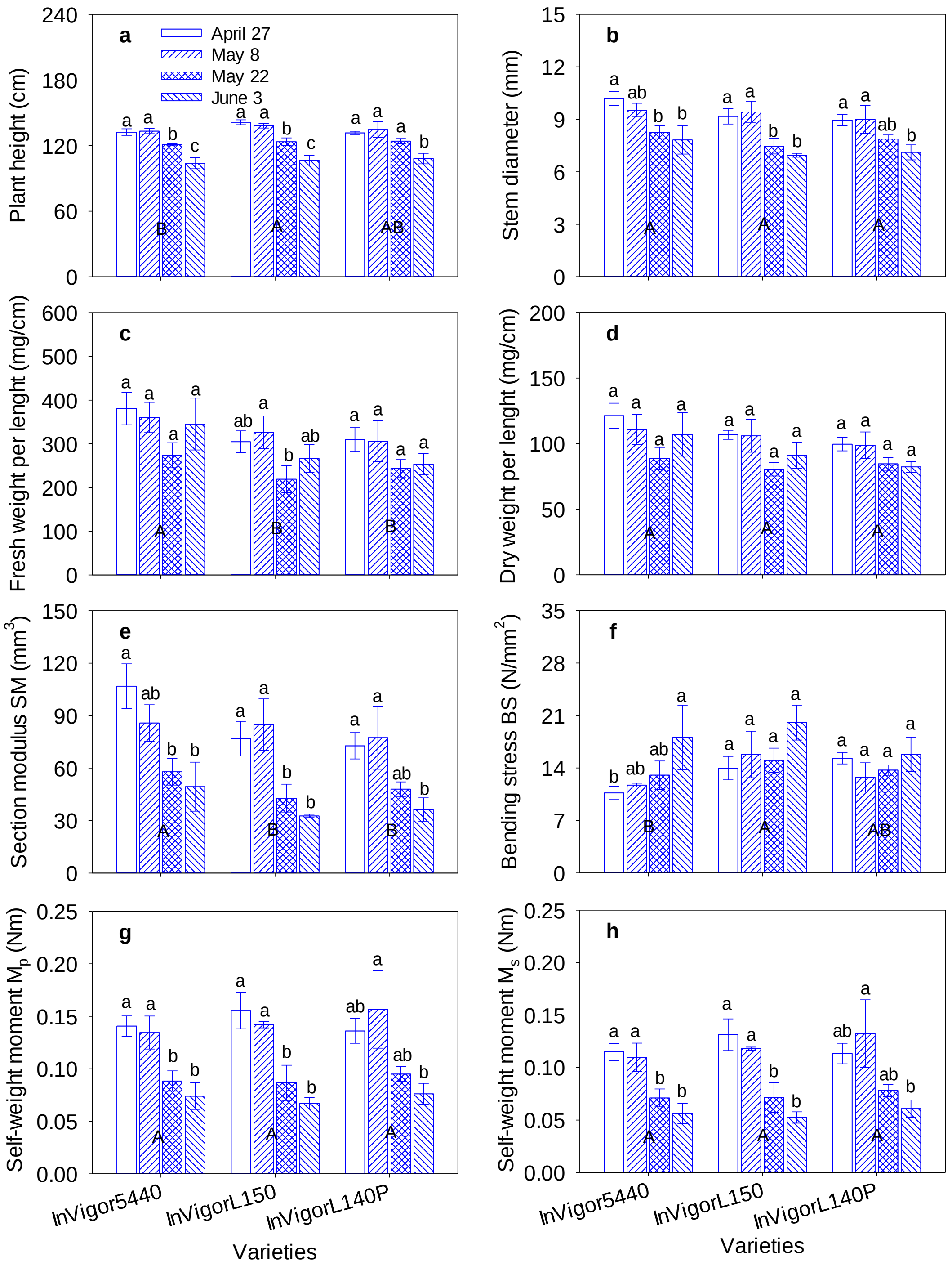

Fig. S4. Effect of sowing date on plant height (a), stem diameter (b), fresh weight per length (c), dry weight per length (d), section modulus (e), bending stress (f), self–weight moment M_p (g) and self–weight moment M_s (h) between three varieties of canola in 2015. Vertical bars above mean values indicate standard error of three replications. Means with different small alphabetical letters show the significant differences between four planting dates for each variety according to LSD (0.05). Means with different capital alphabetical letters show the significant differences between three varieties according to LSD (0.05).

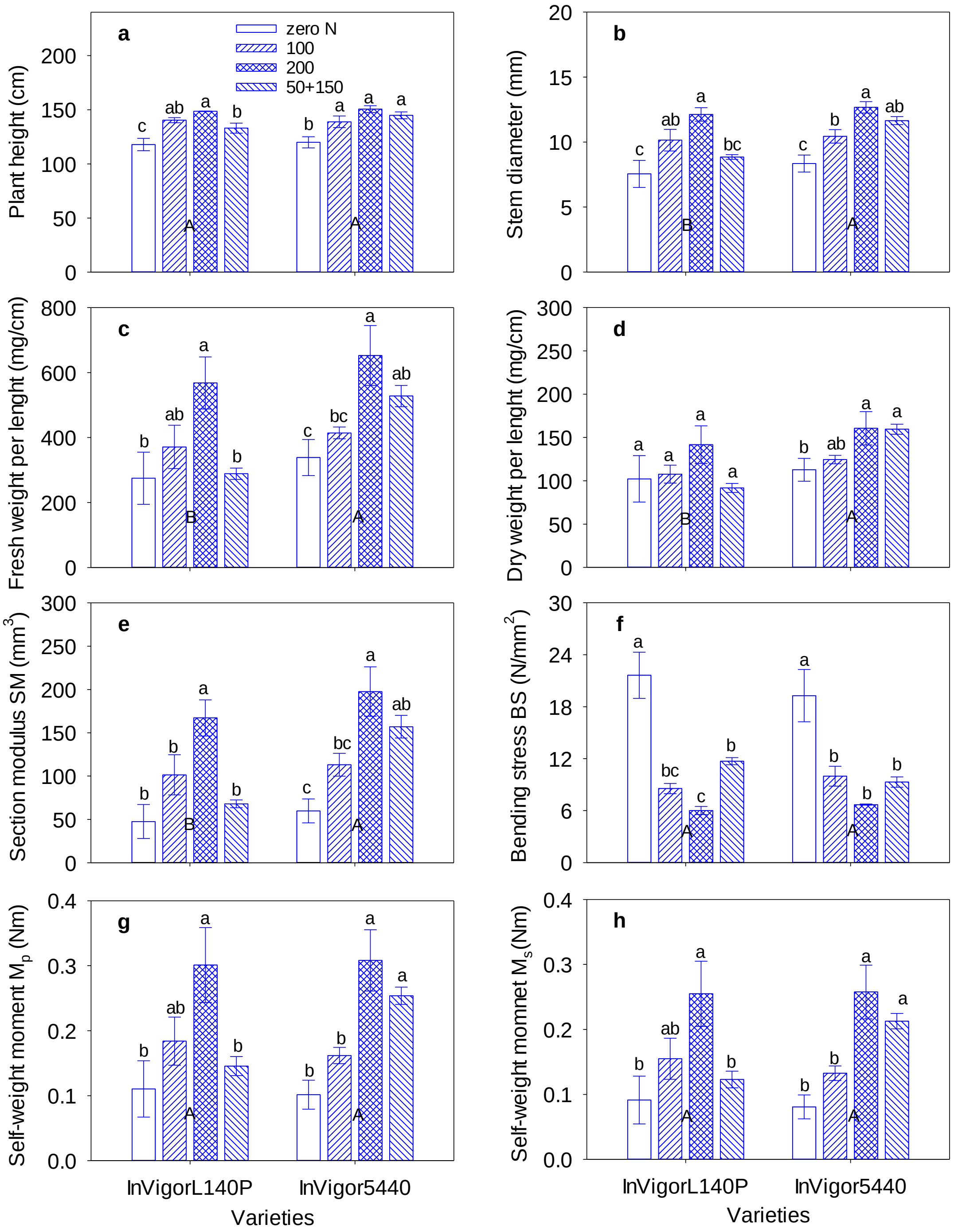

Fig. S5. Effect of N fertilizer management on plant height (a), stem diameter (b), fresh weight per length (c), dry weight per length (d), section modulus (e), bending stress (f), self–weight moment M_p (g) and self–weight moment M_s (h) between two varieties of canola in 2015. Vertical bars above mean values indicate standard error of three replications. Means with different small alphabetical letters show the significant differences between four N fertilizer managements for each variety according to LSD (0.05). Means with different capital alphabetical letters show the significant differences between two varieties according to LSD (0.05).

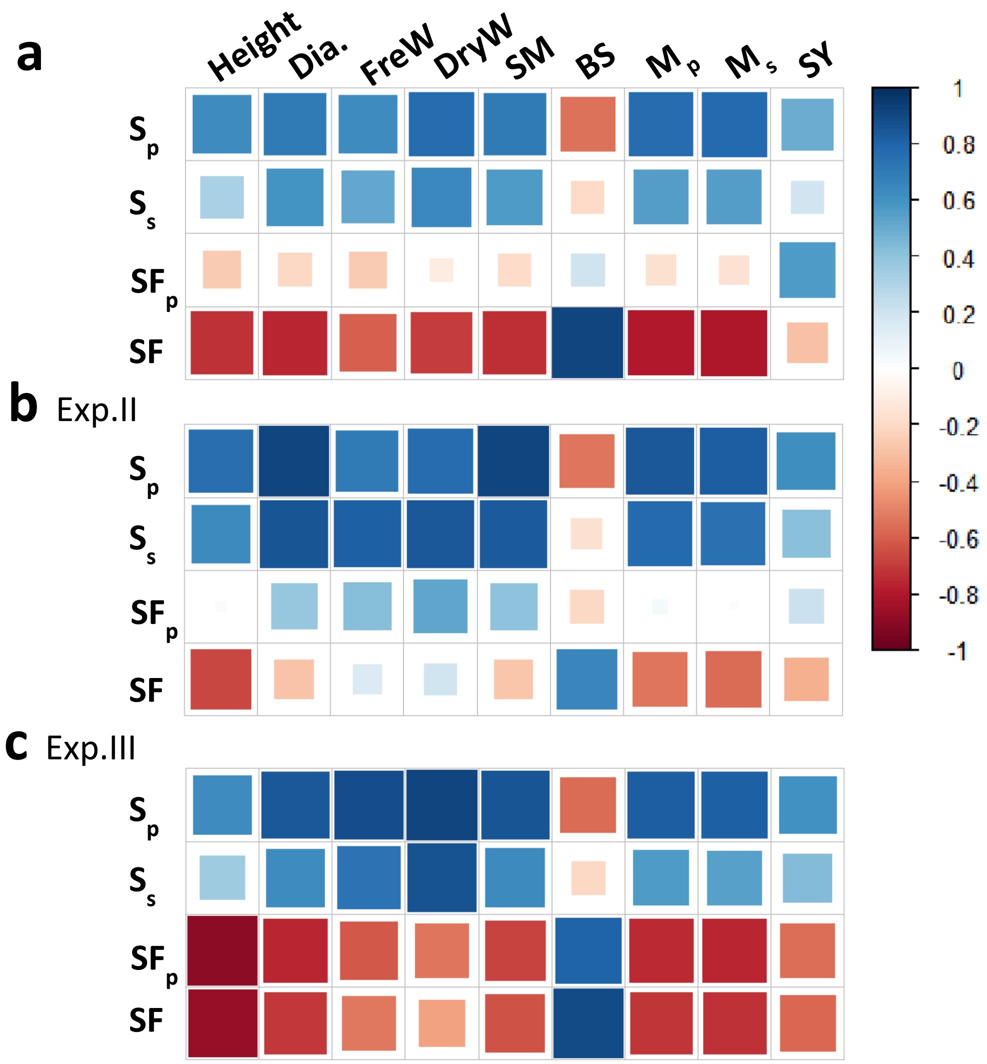

Fig. S6. Colour map for the relationship of four important lodging–related parameters (S_p, root anchorage strength, S_s: stem bending strength, SF_p: root safety factor, SF_r: stem safety factor) with plant height, basal stem diameter (Dia.), fresh weight per length (FreW), dry weigh per length (DryW), section modulus (SM), bending stress (BS), self–weight moment for root (M_p), self–weight moment for stem (M_s) and seed yield (SY). High colour density and larger square area indicates strong relationship. Blue and red colour represents positive and negative relationship, respectively

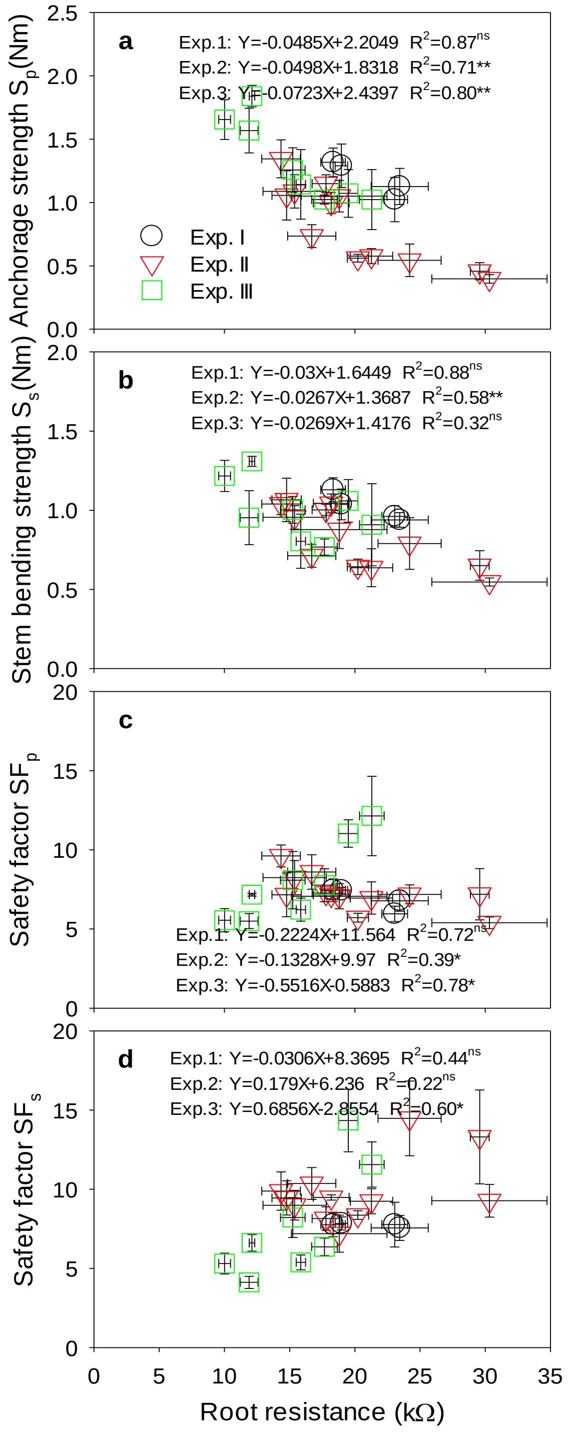

Fig. S7. Relationship of root resistance with anchorage strength (a), stem bending strength (b), root safety factor SF_p (c) and stem safety factor SF_s (d) among three field experiments in 2015. ** indicates significant at $p \leqslant 0.01$; * indicates significant at $p \leq 0.05$; ns indicates non-significant.

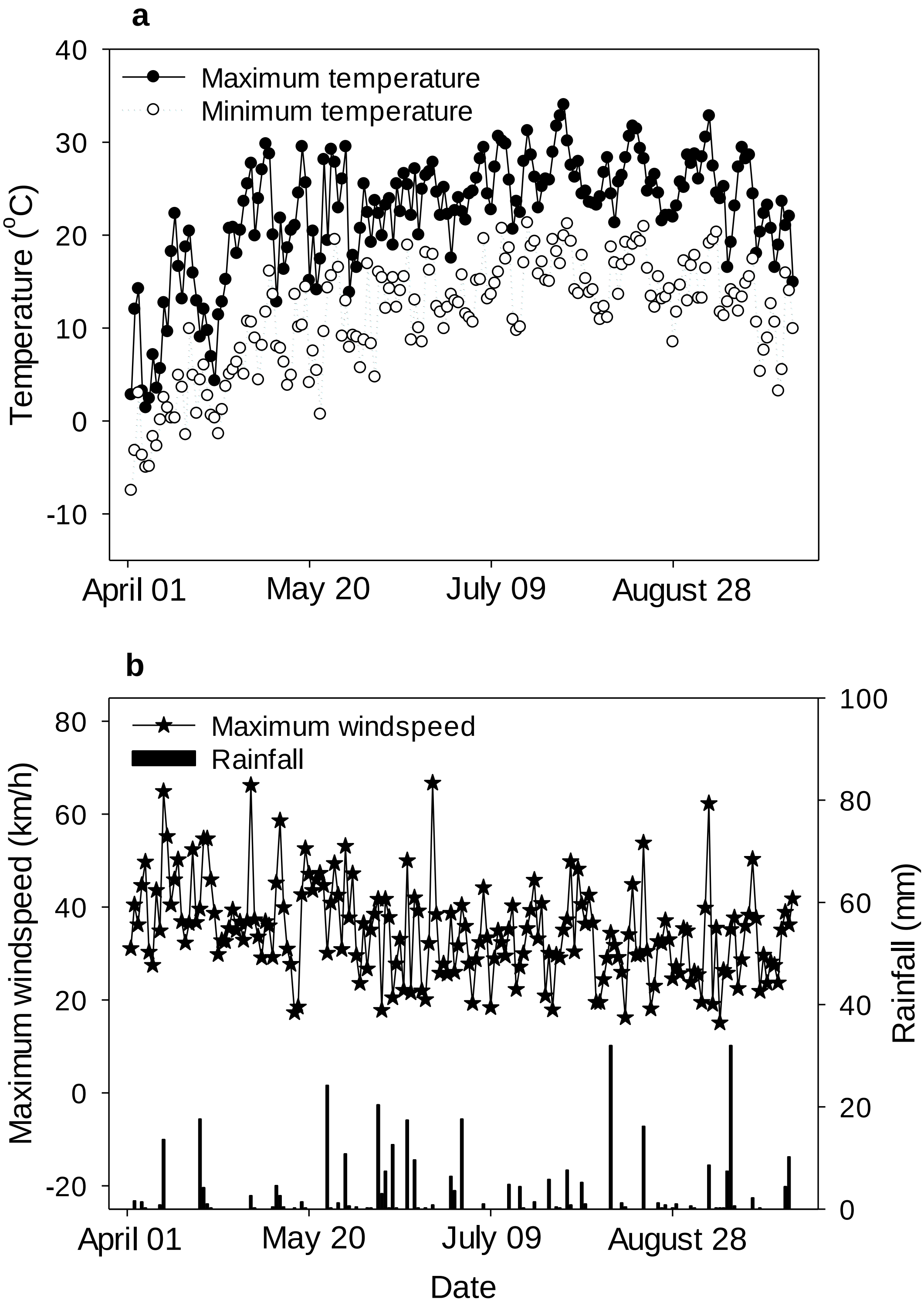

Fig. S8. Daily maximum temperature, minimum temperature, maximum windspeed, and rainfall during the growing season in 2015 at Central Experimental Farm of Agriculture and Agri–Food Canada, Ottawa, ON

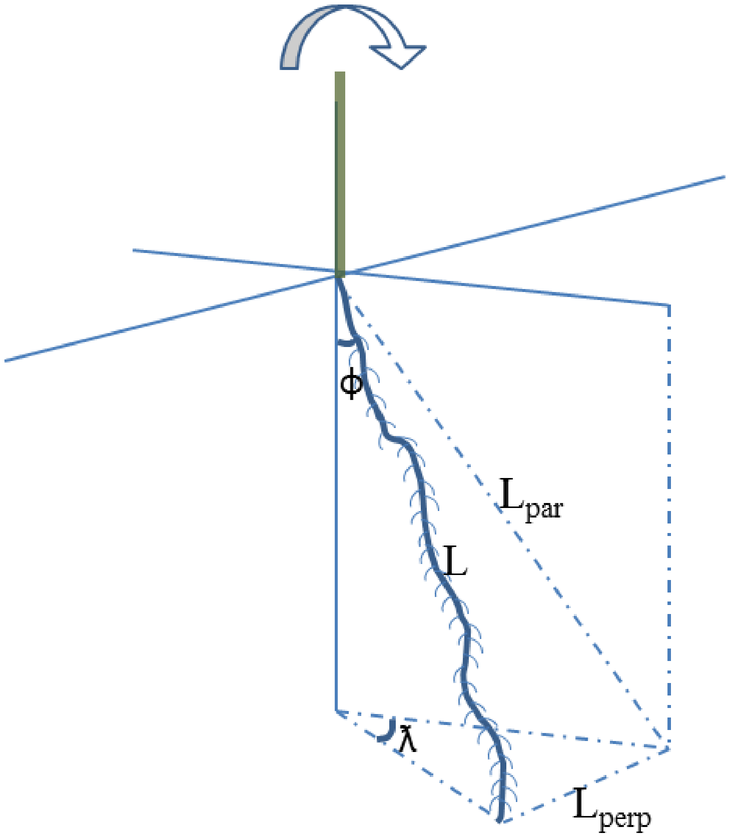

Fig. S9. Diagram showing the geometrical relationships between the root length (L) and projected length of a lateral root along the plane of lodging (L_{par}), and projected length from vertical of the plane of lodging (L_{perp}) during the root–soil cone rotation. The root has length of L, and is orientated at an angle of ϕ, to the vertical and at an angle of ƛ, to the cone of root lodging. This Figure is revised according to Ennos et al. (1991)

Table S1. Analysis of variance for all measurements including root capacitance (C), resistance (R), impedance (Z), root length per plant (L), surface area per plant (A), volume per plant (V), root dry weight per plant (DryR), plant height (H), height of the centre of gravity of total plant (h_p), height of the centre of gravity of stem (h_s), basal stem diameter (Dia), fresh weight of 20 cm basal internode per length (FreW) and dry weight of 20 cm basal internode per length (DryW), section modulus (SM), bending stress (BS), self–moment for total plant (M_p), self–moment for stem (M_s), root anchorage strength (S_p), stem bending strength (S_s), root safety factor (SF_p), stem safety factor (SF_s) and seed yield (SY) across all treatments for the three experiments in 2015

Source	C	R	Z	L	A	V	DryR	H	h_p	h_s	Dia	FreW	DryW	SM	BS	M_p	M_s	S_p	S_s	SF_p	SF_s	SY
Experiment I																						
Irrigation (I)	*	ns	ns	ns	*	*	*	ns	ns	ns	ns	ns	ns	ns	ns	ns	ns	ns	*	ns	ns	ns
Variety (V)	ns	ns	ns	*	ns	ns	ns	ns	ns	ns	ns	ns	ns	ns	ns	ns	ns	ns	ns	ns	ns	ns
I × V	ns	ns	ns	ns	ns	ns	ns	ns	ns	ns	ns	ns	ns	ns	ns	ns	ns	ns	ns	ns	ns	ns
Experiment II																						
Planting data (PD)	**	**	**	-	-	-	-	*	*	*	**	*	*	**	ns	**	**	**	**	*	ns	**
Variety (V)	ns	*	*	-	-	-	-	ns	ns	ns	ns	*	ns	ns	ns	ns	ns	ns	ns	*	ns	ns
PD × V	ns	ns	ns	-	-	-	-	ns	ns	ns	ns	ns	ns	ns	ns	ns	ns	ns	ns	ns	ns	ns
Experiment III																						
Fertilizer (F)	**	**	**	-	-	-	-	**	**	**	**	*	ns	*	**	ns	**	*	ns	*	**	**
Variety (V)	**	**	**	-	-	-	-	ns	ns	ns	**	*	**	**	ns	ns	ns	ns	*	ns	**	ns
F × V	**	**	**	-	-	-	-	ns	ns	ns	ns	ns	ns	ns	ns	ns	*	ns	ns	ns	ns	ns

Levels of significance indicated: ns = not significant, * significant at p≤0.05, **significant at p≤0.01.

Table S2. The explanations for abbreviations

Abbrev.	Explanations	Abbrev.	Explanations
SDI	subsurface drip irrigation	M_p	self–moment for total plant (Nm)
N	nitrogen	M_s	self–moment for shoot (Nm)
C	root capacitance (nF)	S_p	root anchorage strength (Nm)
R	root resistance (kΩ)	S_s	stem bending strength (Nm)
Z	root impedance (kΩ)	SF	safety factor; a dimensionless parameter
L	root length per plant (cm)	SF_p	safety factor against anchorage failure
A	root surface area per plant (cm^2)	SF_s	safety factor against stem buckling
V	root volume per plant (cm^3)	τ	soil shear strength ($N\ m^{-2}$)
DryR	root dry weight per plant (g)	SY	seed yield per plant ($g\ plant^{-1}$)
H	plant height (cm)	ρ_{root}	the electrical resistivity of the root interface
h_p	height of the centre of gravity of total plant (cm)	ξ	the dimensionless coefficient assessing the effect on mutual electric shielding of roots
h_s	height of the centre of gravity of shoot (cm)	K	dimensionless constant, to link τ and root–soil cone diameter with S_p
g	the acceleration owing to gravity ($N\ kg^{-1}$)	M_b/m_b	root resistance to being bent strength (Nm)
Dia/D/r	diameter (cm)	M_a	root resistance to axial motion strength (Nm)
FreW	basal fresh weight of 20 cm internode per length ($g\ cm^{-1}$)	$\phi, \lambda, \beta, \alpha$	angle of inclination
DryW	basal dry weight of 20 cm internode per length ($g\ cm^{-1}$)	F/f	maximum force (N) withstanding before fails
SM	section modulus for basal stem (mm^3)	k	the reciprocal of the distance (mm) between the supports in the three–point bending test
m_s	fresh weight of shoot	ε_i	dielectric constant; a ratio of the permittivity of a substance to the permittivity of free space
m_p	fresh weight of total plant (g)	ζ	the dimensionless coefficient that indicates a negative error in assessing absorbing root surfaces caused by electric current flowing
BS	bending stress of basal stem (N/mm^2); a mechanical parameter for lodging resistance that is influenced by the chemical composition of the culm	η	the dimensionless coefficient that represents the effect of the possible mechanical injury of roots

Table S3. Soil chemical properties of the top 0–30 cm layer in three field experiments in 2015 at Central Experimental Farm of Agriculture and Agri–Food Canada, Ottawa, ON

Parameters	Exp. I	Exp. II	Exp. III
Soil classification	Uplands sand	North Gower clay loam	North Gower clay loam
SOM[a] (g kg^{-1})	4.0	3.8	4.3
pH	6.5	6.7	7.2
Total CEC[b] (meq kg^{-1})	70	190	220
Available P (ppm)	47	45	35
Available K (ppm)	60	190	240
Available Ca (ppm)	1300	2800	3400
Available Mg (ppm)	110	560	460
Available Na (ppm)	10	30	30

[a]SOM = Soil organic matter; [b]CEC= Cation exchange capacity
